# The impact of molecular tumor profiling on the design strategies for targeting myeloid leukemia and EGFR/CD44-positive solid tumors

**DOI:** 10.3762/bjnano.12.31

**Published:** 2021-04-29

**Authors:** Nikola Geskovski, Nadica Matevska-Geshkovska, Simona Dimchevska Sazdovska, Marija Glavas Dodov, Kristina Mladenovska, Katerina Goracinova

**Affiliations:** 1 Institute of Pharmaceutical Technology, Faculty of Pharmacy, University of Ss. Cyril and Methodius in Skopje, Skopje, North Macedonia; 2 Center for Pharmaceutical Biomolecular Analyses, Faculty of Pharmacy, University of Ss. Cyril and Methodius in Skopje, Skopje, North Macedonia; 3 Department of Nanobiotechnology, Institute of Biotechnology, Brandenburg University of Technology Cottbus-Senftenberg, Senftenberg, Germany; 4 College of Pharmacy, Qatar University, PO Box 2713, Doha, Qatar

**Keywords:** CD44, EGFR, liquid tumors, molecular tumor targeting, myeloid leukemia, solid tumors, surface-engineered nanoparticles

## Abstract

Nanomedicine has emerged as a novel cancer treatment and diagnostic modality, whose design constantly evolves towards increasing the safety and efficacy of the chemotherapeutic and diagnostic protocols. Molecular diagnostics, which create a great amount of data related to the unique molecular signatures of each tumor subtype, have emerged as an important tool for detailed profiling of tumors. They provide an opportunity to develop targeting agents for early detection and diagnosis, and to select the most effective combinatorial treatment options. Alongside, the design of the nanoscale carriers needs to cope with novel trends of molecular screening. Also, multiple targeting ligands needed for robust and specific interactions with the targeted cell populations have to be introduced, which should result in substantial improvements in safety and efficacy of the cancer treatment. This article will focus on novel design strategies for nanoscale drug delivery systems, based on the unique molecular signatures of myeloid leukemia and EGFR/CD44-positive solid tumors, and the impact of novel discoveries in molecular tumor profiles on future chemotherapeutic protocols.

## Introduction

The conventional chemotherapy regimens of both liquid (hematological) and solid tumors are challenged by their lack of targeting ability, which usually results in therapy failure and noticeable side effects. Nanomedicine has emerged as a novel cancer treatment and diagnostic modality, whose design constantly evolves towards increasing the safety and efficacy of the chemotherapeutic and diagnostic protocols. Recently, a novel generation of smart nanoscale drug delivery carriers with increased selectivity and multistage targeting capabilities has emerged. Common cancer signatures and the synthesis of ligands with high avidity for the overexpressed cancer cell receptors are a valuable addition to the general targeting concepts. Important discoveries regarding the surface-expressed receptors and their intracellular molecular pathways have been made during the last decade, which improved the design of targeted anticancer nanomedicines and the targeting of specific tumor types. No doubt that the advances will further progress to the application of individualized tumor signatures for a personalized therapy against cancers. The greatest interest regarding the development of targeted nanoscale drug delivery systems is related to solid tumors. However, liquid tumor targeting can greatly benefit from the application of nanomedicines during therapy. Unlike solid tumors, which necessitate nanoscale drug delivery system (NDDSs) to reach a specific site of action, liquid tumors are mainly spread throughout the blood circulation. In fact, the barriers that apply to the NDDSs for solid tumor targeting usually do not exist in the case of liquid tumors, since the tumor cells are generally exposed in the blood circulation. Hence, hematological tumors generally require a slightly different approach for diagnosis and treatment. However, the discovery of the leukemic stem cells (LSCs), which are located in the bone marrow endosteal region, has introduced the possibility of employing similar strategies for passive targeting as for solid tumors, but these tumors also have much in common regarding the expression of specific molecules as viable targets for therapy and/or homing of NDDSs. For example, overexpression of the EGFR gene or protein kinase cascades downstream the growth factor receptor mutations, which were detected in a variety of solid tumors, in diverse acute myeloid leukemia (AML) cells, and in many other leukemic cells, highlighted their role as therapeutic targets and the importance of novel therapeutic approaches and treatment alternatives using NDDSs [1]. Literature data points to combinatorial therapy, coadministration, and codelivery of agents by nanomedicines as a successful approach to bypass signaling inhibition, combat anticancer drug resistance, and increase the efficacy of the clinical treatment. Further advances in molecular screening and novel discoveries related to unique cancer molecular signatures, including the specific biomarkers of the cancer stem cells and the correlated action of receptors in cancer survival, such as the frequently reported CD44/EGFR axis, resulted in useful strategies for the development of dual-targeted NDDSs capable of attacking multiple targets for a more efficacious cancer treatment [2]. With this in mind, in this article we will review novel NDDS design strategies, based on the unique morphological and molecular signatures of liquid (myeloid leukemia) and solid tumors. The focus on myeloid leukemia and solid tumors with overexpression of EGFR and/or CD44 was chosen to highlight the differences and similarities of the NDDS design strategies for targeting two seemingly different morphological and molecular types of tumors and to illustrate the impact of novel discoveries in molecular tumor profiles and targeting strategies on the future chemotherapeutic protocols.

## Review

### Molecular profile and characteristics of myeloid leukemia

Leukemia comprises a heterogeneous group of diseases characterized by abnormal proliferation and differentiation of a clonal population of blood progenitor cells. The proliferation of the self-renewing malignant clones develops and expands in the bone marrow from where the cells start to circulate to the peripheral hematopoietic tissues, leading to a severe and often fatal systemic malignancy that affects hematopoiesis, immune defense system, and many other body systems [3]. All types of leukemia account for 2.5% of overall cancer incidence and 3.1% of cancer mortality worldwide. Estimated numbers for new cases and deaths in 2020 are 474,519 and 311,594, respectively [4]. In adults, AML and chronic lymphocytic leukemia (CLL) are the most common types of leukemia. In children aged 0 to 14 years, leukemia is the most commonly diagnosed cancer, accounting for up to 28% of all cancers, of which over 75% are acute lymphoblastic leukemia (ALL) [5].

The treatment depends on the type of leukemia, disease stage, prior history of treatment, age, overall condition, and genetic profile. The current treatment options include traditional chemotherapy, haematopoietic stem cell transplantation (HSCT), and targeted therapy (small molecule inhibitors and monoclonal antibodies). Traditional chemotherapeutic regimens are limited by two primary concerns: overall toxicity and lack of efficacy (resistance). Nevertheless, the application of cytotoxic chemotherapeutics (e.g., daunorubicin and cytarabine), with or without HSCT, still remains the backbone of treatment for AML [6–7]. All chemotherapy agents interfere somehow with the DNA replication process or with cell mitosis. Anthracyclines (such as daunorubicin) interfere with topoisomerase II and inhibit DNA replication and histone activity. Alkylating agents (such as cyclophosphamide) introduce inter- and intrastrand cross-linkages and breaks in the DNA. Antimetabolites (such as 5-fluorouracil) obstruct the synthesis of nucleic acids required for DNA replication, while taxanes and vinca alkaloids interfere with the polymerization/depolymerization of the microtubules thus inhibiting mitosis [8]. However, in addition to cancer cells, all of these drugs also affect the normal/healthy tissues with cells that rapidly divide, such as bone marrow stem cells and the gastrointestinal epithelium. This leads to severe and sometimes life-threatening side effects and disruption of the normal hematopoiesis and subsequent recovery, especially of elderly patients.

The lack of sensitivity and the development of resistance is another major drawback of the traditional “broad-spectrum” cytotoxic drugs. Leukemic cells generally respond well to drug therapy at the onset of treatment, but the drugs lose their effectiveness over a period of 6–12 months in a significant fraction of patients [9]. In contrast to solid tumors, where cancerous cells accumulate at defined tumor sites and the cytotoxic drugs could be passively targeted to these tumor sites through enhanced permeation and retention (EPR) effect, leukemic cells are prevalent in the whole circulatory system and the EPR is of no use. Moreover, the inherent plasticity of the leukemic cells combined with diverse resistance mechanisms allows malignant cells to naturally adapt to drugs. Several resistance mechanisms have been acknowledged, including failure of the cell to undergo chemotherapy-induced apoptosis and failure of the drug to reach and/or affect its intracellular target due to intracellular drug transport resistance mechanisms [10]. Additionally, the high recurrence rate in leukemia patients has been also attributed to the existence of a rare population of LSCs capable of evading drug therapies [11–12]. These CD34^+^CD38^−^ LSCs, which preferentially reside in the bone marrow endosteal region, have acquired abnormal self-renewal and have shown the capability to give rise to heterogeneous nonstem leukemic cells. Therefore, they are considered responsible for disease initiation and maintenance. Moreover, the CD34^+^CD38^−^ LSCs predominantly exist in the quiescent phase of the cell cycle (G0). They, hence, have a resistance to cytotoxic drugs that interfere with cell mitosis [11–12]. These observations imply that minimal residual disease (MRD) can be attributed to rare quiescent CD34^+^CD38^−^ LSCs remaining after therapy and highlight the importance of the complete eradication of the LSCs in improving the long-term outcomes in leukemia patients. This suggests the need for the identification of LSC-specific molecules, which can be employed as drug targets for the development of novel therapeutic antibodies and inhibitors of LSC-specific kinases or transcription factors, but also as roadmaps for active targeting using novel nanoparticles (NPs). Furthermore, the use of nanotechnology for the delivery of cytotoxic drugs can also be valuable in facilitating cell-specific administration of drugs, improving their bioavailability, reducing side effects, and restoring the efficacy/response, especially through evading the drug transport resistance mechanisms.

In contrast to traditional cytotoxic chemotherapy, targeted therapies, such as tyrosine kinase inhibitors (TKIs), are directed towards the molecular aberrations responsible for elevated kinase activity or towards fusion oncoproteins involved in proliferative or anti-apoptotic signaling pathways [13]. For instance, more than 90% of the cases of chronic myelogenous leukemia (CML) are characterized by a unique chromosomal abnormality known as Philadelphia (Ph) chromosome [14]. This abnormality is a reciprocal chromosomal translocation between the long arms of chromosomes 9 and 22, designated as t(9;22)(q34;q11), creating a derivative 9q+ and a shortened 22q Ph chromosome. The chimeric Ph chromosome generates the BCR-ABL1 gene by fusion of the Abelson murine leukemia (ABL1) gene from chromosome 9 with the breakpoint cluster region (BCR) gene from chromosome 22. Due to different possible breakpoints on chromosome 22, several transcripts can originate from this translocation. However, all BCR-ABL1 gene fusions described so far encode for a constitutively active BCR-ABL1 tyrosine kinase that promotes growth and replication through downstream pathways such as RAS, RAF, JUN kinase, MYC, and STAT. This influences leukemogenesis by causing enhanced proliferation, differentiation arrest, and resistance to cell death. The use of the TKIs, the first of which was imatinib, has improved the three-year survival to over 95%, from 50% in the pre-imatinib era [7]. However, imatinib and related TKIs (the second-generation TKIs dasatinib, bosutinib, nilotinib, and the third-generation ponatinib) are not exclusively specific for the BCR-ABL fusion protein, and may also affect normal c-ABL and other kinases such as c-KIT. This can lead to side effects, mainly diarrhea and skin toxicity [15]. Also, all TKIs have short half-lives (in the case of imatinib and its main metabolite 18 and 40 h, respectively) and require daily dosing.

More importantly, similar to the traditional cytotoxic agents, resistance to TKIs and early relapse are still major concerns. For example, 15% to 40% of CML patients will develop resistance or intolerance to first-line imatinib. The resistance mechanisms can either depend on BCR-ABL1 or not. BCR-ABL1-dependent resistance mechanisms are primarily associated with the overexpression of the BCR-ABL1 oncogene or mutational events [16]. The clinically relevant mutations arise in the kinase domain of BCR-ABL1 and lead to impaired TKI activity, mainly by preventing the fusion protein from adopting the correct conformation required for specific binding. More than 40 different point mutations have been identified in relapsed CML patients receiving imatinib. However, the most frequent alterations occur in three different regions and seven specific residues, that is, in the docking site for phosphate moieties of ATP named P-loop (M244V, G250E, Y253F/H, and E255K/V), in the contact site related to selective BCR-ABL1 inhibition by IM (T315I), and in the catalytic domain (M351T and F359V) [17]. In general, second-generation TKIs are active against most of the subclones resistant to imatinib mesylate (IM), except against the subclones harboring the T315I mutation. The T315I mutation displays resistance to all currently available TKIs, except for ponatinib, a third-generation TKI. In addition, second-generation TKIs also display similar resistance mechanisms as imatinib, but the mutation spectra are different: T315I, F317L or V299L for dasatinib, E255K/V, T315I, F359C/V or Y253H for nilotinib, and V299L or T315I for bosutinib.

#### Molecular targeting of myeloid leukemia using surface-engineered nanoparticles

**Nanomedicines as an efficient tool for bone marrow targeted delivery of drugs:** It is known that solid tumors reside in extravascular spaces. In order to reach them, the NDDSs need to extravasate the porous tumor vasculature. In contrast, leukemic cells and leukemia stem cells are settled in the blood vessels and bone marrow (BM) and seem readily available to the intravenously administered NDDSs. Nevertheless, the abundance of normal cell populations in the blood and BM imposes the necessity of a targeted and potent approach in the design of NDDSs for leukemia treatment.

The cancer stem cells, with their self-renewal and tumor-initiating properties, are considered as one of the prime factors affecting the promotion and relapse in most cancer types. As described in the previous section, the enhanced DNA repair ability, the expression of anti-apoptotic proteins, and the drug efflux transporters of the leukemia stem cells are considered the main contributors to the resistance to chemo- and radiotherapy of many types of leukemia. Hence, it is logical to identify this cell population as a prime target for leukemia therapy.

The BM microenvironment provides a protective accommodation for the normal stem cells, and many studies suggest that the leukemia stem cells use the same shelter for their own survival. Therefore, targeting leukemia cells located in the BM is not a straightforward issue regarding intravenously administered NDDSs. The BM is a primary lymphoid tissue that is located inside various bone types. It can be classified as red or yellow marrow, depending on whether the dominant cell population is of hematopoietic origin or fat cells. There are two types of stem cells in the BM, hematopoietic, which are responsible for the hematopoiesis, and mesenchymal, which produce the stromal, fat, cartilage, and bone tissue. The BM stroma contains fibroblasts, macrophages, adipocytes, osteoblasts, osteoclasts, and endothelial cells. It provides the appropriate microenvironment for efficient hematopoiesis. The blood flow is supported by the nutrient arteries, which supply the marrow with nutrients, the periosteal artery, arterioles, and the capillary circulation, which forms a sinusoidal network of vessels comprising a single endothelial cell layer lacking supporting cells. The barrier between the hematopoietic compartment and the blood circulation is commonly referred to as the marrow–blood barrier (MBB). The MBB is 2–3 µm thick. It is composed of the continuous vascular endothelium and the discontinuous adventitial reticular cell layer. Hence, it is relatively highly permeable to a wide variety of solutes and even particulate matter [18]. Portions of the sinusoidal endothelial cells could be noticeably reduced into small fenestrae with sizes in the range of 80–150 nm, which could facilitate the paracellular MBB transport. On the other hand, their clathrin-coated pits, lysosomes, clathrin-coated vesicles, and phagosomes could be employed as a gateway for endocytosis-mediated transcellular transport across the MBB [19]. Since there is no lymphatic drainage, the BM tissue relies on these pathways for bidirectional transport of a variety of molecules across the MBB. The capability of the sinusoidal endothelial cells, and the present fenestrae, to remove colloid particles from the bloodstream nurtures the possibility of targeting the leukemia stem cells with NDDSs.

**Figure 1:**
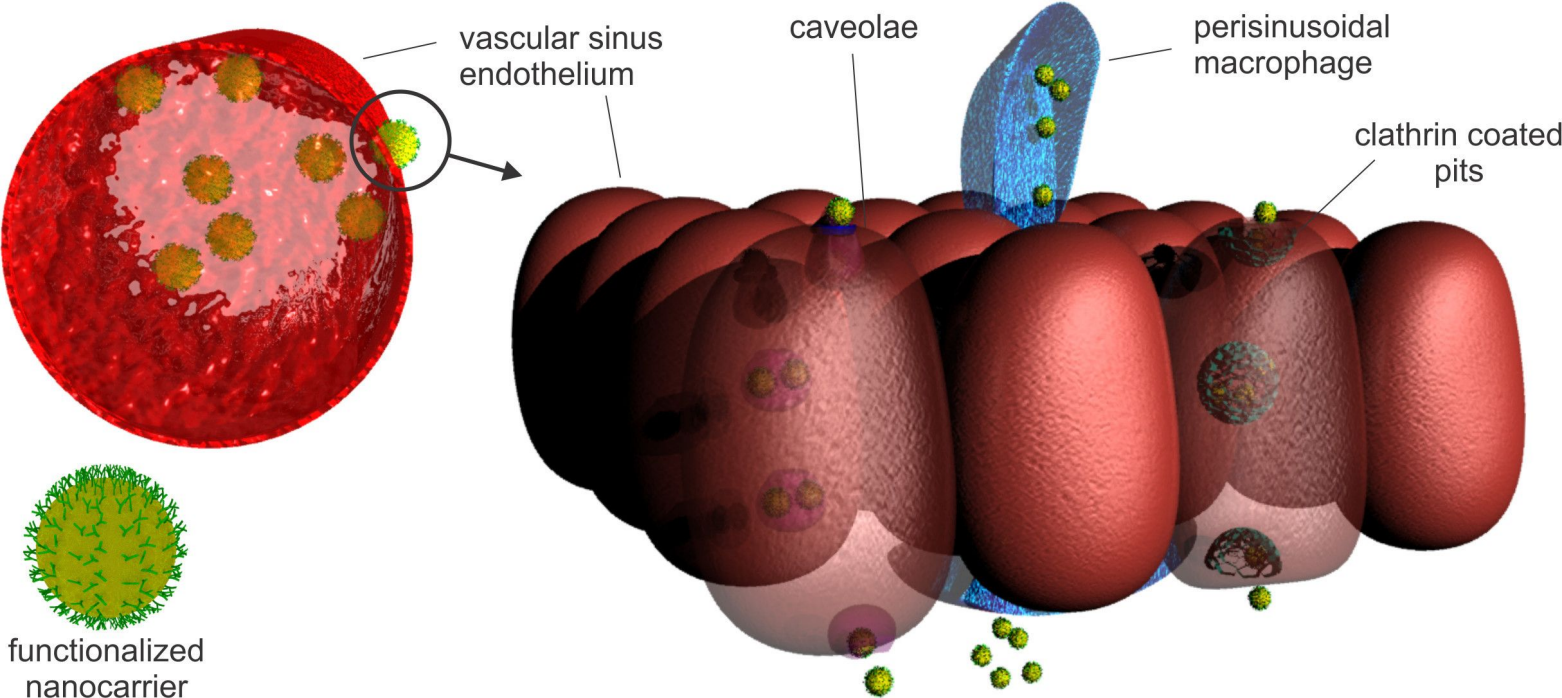
Hypothetical mechanisms of extravasation of NDDSs into the BM stroma. The nanoscale carrier could induce clathrin- or caveolin-mediated endocytosis into the vascular sinus endothelium, resulting in transcytosis towards the BM stroma. Another possible mechanism of extravasation of NDDSs is the uptake mediated through perisinusoidal macrophages.

In order to reach the BM vasculature, it is of utmost importance that the NDDSs do not interact with the elements of the reticuloendothelial system (RES) located in the liver and spleen. Such interactions will result in RES-induced sequestration of the NDDSs, drastically reducing its availability in the organ of interest (BM). Additionally, to effectively extravasate, a circulating NDDS firstly needs to drift to the margins of the blood vessels, and then recognize the specific endothelial target to which it needs to firmly adhere. Afterward, the adhered NDDS should induce endocytosis-mediated internalization into the BM vascular sinus endothelium, which probably will result in carrier transcytosis towards the stroma [20] (Figure 1).

Illum and Davis were among the first researchers that reported on the possibility of guiding colloidal particles to the BM [21]. The initial goal of the group was to evaluate the effects of different PEO–PPO-based hydrophilic copolymer (poloxamer) shells upon the biodistribution, primarily the circulation half-life of polystyrene colloidal particles [21–22]. In their experiments, besides the groundbreaking work on the steric barrier and the so-called “stealth” effect of the poloxamer, they have further noticed that the adsorbed poloxamer 338 and 407 shells directed the polystyrene beads to the sinusoidal endothelial cells of rabbit BM [23]. Even though the authors eliminated the possibility of a BM distribution mediated through macrophage phagocytosis, the exact mechanism of localization remained unknown. Moreover, the poloxamer-coated polystyrene beads circulated in the blood for up to eight days, and regardless of the steric barrier, somehow managed to interact with the sinusoidal endothelial cells of the BM. One possible explanation is that the recognition may be mediated by a plasma component (such as erythropoietin, transferrin, or transcobalamin) or an endothelial factor that specifically adsorbs onto the surface of poloxamer 407-coated colloidal particles, thus exhibiting microdomains that are specific for the sinusoidal BM endothelium.

Another mechanism of BM targeting is the phagocytosis-mediated uptake from the perisinusoidal macrophages. It is known that the perisinusoidal BM macrophages protrude through the vascular endothelial wall to gain access and monitor blood circulation [24]. Hussain et al demonstrated that perisinusoidal macrophages are responsible for the accumulation of the chylomicrons in the BM and, hence, play a crucial role in the delivery of lipids, as a source of energy and material for membrane biosynthesis, and fat-soluble vitamins [25]. In the past, this pathway was generally ignored as a targeting possibility, mostly because the BM contribution to the RES is quite negligible compared to that of spleen and liver. Also, there was a lack of understanding and knowledge regarding the presence of any specific BM macrophage moieties. Considering the aforementioned, the development of an effective targeting system will rely on strategies that will enable evasion of liver and spleen uptake and, at the same time, facilitate BM uptake using appropriate ligands.

BM-targeting liposomes were among the first formulation types that employed this specific mechanism. Allen et al. reported that the lipid composition greatly affected the bone marrow affinity of the liposomes. The authors noted that phosphatidylserine and phosphatidylcholine increased the uptake of the liposomes in cultured BM macrophages [26]. Schettini et al. made an attempt at BM targeting by incorporating negatively charged lipids in the liposomes. However, the employed lipid components (1,2-distearoyl-*sn*-glycero-3-phosphocholine, cholesterol, and dicetylphosphate) did not promote selective BM uptake [27]. Moreover, the authors reported that the size reduction of the liposomes produced better results. The surface modification of liposomes with an anionic amphiphile (*N*-(3-carboxy-1-oxopropyl)-ʟ-glutamic acid 1,5-bis(hexadecyl ester)), reported by Sou and co-workers, resulted in significant improvement in BM uptake. The authors noted that, besides the anionic amphiphile, a small amount of 1,2-distearoyl-*sn*-glycero-3-phosphoethanolamine-poly(ethylene glycol) (DSPE-PEG) was needed to circumvent the liver and spleen sequestration and to achieve a distribution of 60% of the injected liposomes inside the BM within 6 h after administration [28]. The addition of larger quantities of surface-oriented DSPE-PEG resulted in a reduction of BM uptake, probably due to steric hindrance of the anionic amphiphile, which is considered as the active targeting moiety. In their further research, the authors confirmed that the BM uptake mechanism relies on phagocytosis-mediated lipid transport. In fact, when administered in higher doses or along with other specific lipids, the BM macrophages get saturated such that the phagocytosis rate drops resulting in a cessation of BM transport of the liposomes [29]. Tardi and co-workers developed distearoylphosphatidylcholine/distearoylphosphatidylglycerol/cholesterol (DSPC/DSPG/Chol) liposomes with BM targeting capability [30]. The synthesized liposomes were able to maintain the BM levels of cytarabine and daunorubicin for longer periods and demonstrated, respectively, threefold and eightfold higher BM drug concentrations relative to the plasma concentration in the 48th hour post-administration in healthy mice. In contrast, the combination of free drugs (cytarabine and daunorubicin) reached maximum BM concentrations in one hour, which decreased rapidly. The authors estimate that the high levels of BM accumulation for this liposome formulation were probably due to the presence of 20% DSPG in the liposomal bilayer. This yields an overall anionic surface, which is known to have an affinity for the scavenger receptor on BM macrophages. Other polyanions, such as dextran sulfate, were used as ligands for targeting this macrophage receptor [31]. Yet, not all polyanions could be considered as viable ligands for BM macrophage targeting [32].

Another mechanism of BM targeting was reported by Swami et al. who employed the specific affinity of bisphosphonates for bone tissues with higher turnover rates [33]. The authors used alendronate as a surface-exposed ligand for directing bortezomib-loaded nanoparticles consisting of poly(ᴅ,ʟ-lactic-*co*-glycolic acid) (PLGA) and poly(ethylene glycol) (PEG) to the bone microenvironment, which resulted in a 9.6-fold increase of bone localization of the functionalized nanoparticles relative to the non-functionalized nanoparticles. Additionally, the loaded PLGA–PEG–alendronate nanoparticles demonstrated satisfactory results in myeloma growth inhibition and overall survival of the murine models. Considering the toxicity of bortezomib, acquiring this targeted approach could enable the employment of higher doses that will result in a more effective therapy with fewer side effects.

In vivo imaging of the epithelium of BM blood vessels revealed that the vasculature expresses the adhesion molecule E-selectin and the chemoattractant stromal-cell-derived factor 1 in discrete, discontinuous areas that influence the homing ability of a variety of tumor cell lines inside the BM, and that these specific molecules determine a microenvironment for early BM tumor metastasis [34]. This specific feature was employed by Mann and co-workers, who developed E-selectin-targeted porous silicon particles with capabilities for selective uptake to the BM [35]. The authors formulated a multistage carrier composed of porous silica microparticles that encapsulate nanoscale paclitaxel-loaded liposomes. The porous silica particles were decorated with E-selectin thioaptamer ligand (ESTA), which binds to E-selectin with high affinity and at the same time expresses minimal cross-reactivity to other selectin family members [36]. The formulation demonstrated an eightfold increase in the BM localization of the encapsulated liposomes, relative to the equivalent without ligands, in a healthy murine model. Even though the authors reported an organ distribution of nearly 20% per gram in the BM, most of the porous silicon particles were entrapped in the spleen, liver, and lungs, because of their size and surface characteristics. In this case, the physicochemical properties of the carrier dominate the overall biological behavior of the NDDS, although some specificity was achieved through the employment of a specific targeting ligand.

**Strategies for targeting specific leukemia cells using NDDS:** As discussed in the previous section, nanotechnology enables selective delivery of a wide variety of therapeutic molecules to cancer cells. This allows the molecules to reach critical tissue compartments, such as the BM and lymph nodes, which are otherwise inaccessible to the drugs. Targeting specific surface-exposed moieties of the cancer cell subpopulations is considered as a general strategy of active targeting. It could be essential in leukemia treatment, especially in the cases where a persistent clone dominates the leukemia cell population. Taking into account the molecular profile of the disease, there are a plethora of overexpressed molecules in leukemia cancer cells, which could be used as potential targets for a NDDS-based therapeutic approach (Table 1).

**Table 1:** NDDSs in molecular targeting of myeloid leukemia.

type of leukemia	NP type	payload	target	targeting ligand	efficacy assessment	ref.

AML						

	generation 7 poly(amidoamine) (PAMAM) nanoscale dendriplex	miR-150	FMS-like tyrosine kinase 3 (FLT3)	FLT3 peptide	in vitro, in vivo	[37]
	lipopolyplex NPs	antagomiR-126	miR-126	transferrin or anti-CD45.2 antibody	in vivo	[38]
	Gold NPs	rapamycin (immobilized using a glutathione linker)	Tim-3 immune receptor and a trafficker for its natural ligand galectin-9	anti-Tim-3-single chain antibodies	in vitro	[39]
	multistage vector (MSV) system of mPEG-PLA micelles in protective degradable porous silicon particles	parthenolide	E-selectin	E-selectin thioaptamer (ESTA)	in vivo	[40]
	mesoporous silica NPs	siRNA	CD44	hyaluronan	in vitro	[41]
	mesoporous silica NPs	daunorubicin	B220 surface marker	anti-B220 antibody	in vitro, in vivo	[42]
	cyclodextrin-based NPs	siRNA	IL-3 receptor α-chain (IL-3Rα), also known as CD123	fragment antigen-binding (Fab) of a monoclonal antibody	in vitro, ex vivo	[43]
	gold NPs	oligonucleotides anti-221 and AS1411	NCL/miR-221/NF-κB/DNMT1 signaling pathway	nuclear localization signal (NLS) peptide	in vitro, in vivo	[44]

CML						

	gold NPs (AuNP@PEG@e14a2)	tyrosine kinase inhibitor imatinib	e14a2 Bcr-Abl1 transcript	single-stranded DNA oligonucleotide	in vitro	[45]
	magnetic NPs	paclitaxel	lectin receptor	lectin glycoprotein	in vitro, in vivo	[46]
	PEG–PLA micelles	tyrosine kinase inhibitor ponatinib and JAK2 inhibitor SAR302503	hydroxyapatite	alendronate	in vitro, in vivo	[47]

multiple myeloma						

	PLGA-*b*-PEG NPs	bortezomib	hydroxyapatite	alendronate	in vitro, in vivo	[33]

The folate receptor is usually overexpressed in all cancer cells and the high frequency (ca. 70%) of folate receptor (FR) β expression in CML and AML relative to normal hematopoietic cells suggests the feasibility of the development of FR β-targeted delivery systems [48–49]. Even though the concept of FR β targeting seems conceptually feasible, the heterogenicity of the FR β expression levels in CML and AML presents a confounding issue, which can be usually resolved by co-treatment with all-trans retinoic acid (ATRA) or other agonists of nuclear receptors for retinoids [50]. Such approach was demonstrated by Pan and co-workers, who formulated doxorubicin (Dox)-loaded DSPE/cholesterol/PEG liposomes decorated with folic acid and evaluated their effects upon leukemia cells both in vitro and in vivo. The authors noticed that the cytotoxicity of folate-functionalized liposomes was greater in FR positive cell lines and that the effect could be blocked through the addition of 1 mM folic acid. Additionally, the authors revealed that the functionalized carriers increased the median survival of the mouse ascites leukemia models, compared to non-functionalized carriers, and that the pre-treatment of the animals with ATRA further improved the efficacy of the FR-targeted liposomes by up to 50% [51].

Since AML and CML proliferation is mainly governed by leukemia stem cells (LSC), there is a rationale to develop nanoparticulated drug delivery systems targeted to this cell population. It is known that the leukemia-propagating cells in murine CALM/AF10-positive AML differ from normal haematopoietic stem cells regarding the surface expression of B220 [52]. To demonstrate the efficacy of targeting a specific leukemia cell population, Mandal and co-workers developed mesoporous silica nanoparticles decorated with an anti-B220 antibody intended for B220^+^LSC targeting [53]. The daunorubicin-loaded targeted nanoparticles demonstrated selective efficacy against B220^+^/Mac1^−^ cells, relative to B220^−^ AML LSCs. In addition, the treated B220^+^/Mac1^−^ cells were injected in immunodeficient mice, and the median time for leukemia onset in the animals was observed for daunorubicin, daunorubicin-loaded non-functionalized nanoparticles, and anti-B220 antibody-functionalized nanoparticles loaded with daunorubicin. The functionalized NPs demonstrated superior results (median time for onset of leukemia = 160 days) relative to the drug and to non-functionalized NPs (19–22 days), most probably because of the increased intracellular concentrations of daunorubicin present in the B220^+^/Mac1^−^ cells. Even though the B220 receptor is not internalized after antibody binding, the authors speculate that the NP-enriched cell membrane promotes some kind of “passive” internalization of the functionalized NPs.

**Figure 2:**
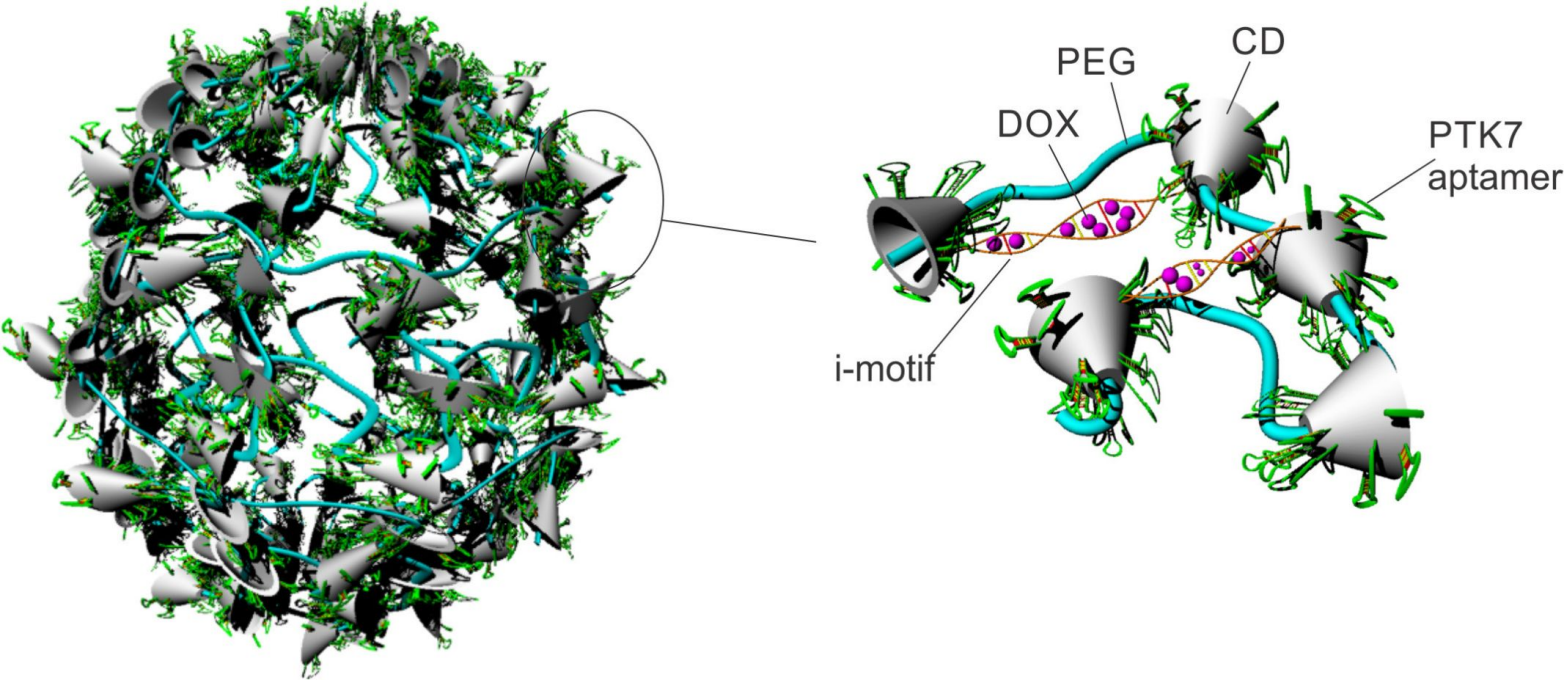
Schematic representation of a polyrotaxane nanoconstruct bearing surface-oriented PTK7 aptamers for targeted delivery of doxorubicin (PEG: poly(ethylene glycol), CD: cyclodextrin, DOX: doxorubicin). The dynamic structure allows for ligand “sliding” for efficient receptor binding and selective DOX release from the DNA i-motifs at lower pH values. Adapted from [55].

Protein tyrosine kinase 7 (PTK7) is a highly expressed receptor in AML cells and is mostly assigned to granulocytic lineage differentiation. Therefore, it could be employed as a specific cell surface-expressed moiety for active targeting [54]. Jang and co-workers developed a polyrotaxane-based nanoconstruct with pliable structure, bearing surface-oriented sliding PTK7 aptamers for the targeting of doxorubicin to a PTK7^+^ cell line (Figure 2). The aptamer DNA–cyclodextrin (CD) component of the polyrotaxan complex demonstrates a high degree of sliding freedom. This unique ability allows for a more efficient binding of the aptamer DNA to target molecules on cancer cells. It simultaneously allows other ligands on the same carrier to migrate toward other target molecules to form multimeric bonds, thus enhancing the robustness of the bonding among the nanoscale carriers and target cells. In addition to the sliding targeting ligands, the authors enabled stimuli-responsive drug release by incorporating i-motif DNA in the nanoscale carrier structure. I-motifs are four-stranded quadruplex structures formed by cytosine-rich DNA that posses unique pH-sensitive characteristics. The double-stranded complementary DNA responds to a drop of the pH value in the environment by forming i-motifs and releasing the intercalated doxorubicin. The targeting potential of the developed formulation was confirmed both with in vitro experiments on the PTK7^+^ T lymphoblast cell line CCRF–CEM and on in vivo immunodeficient BALB/c mice harboring the PTK7^+^ T lymphoblast cell line. The results demonstrated that the sliding targeting aptamers had, respectively, a three and a six times greater affinity for binding the PTK 7 receptor, relative to the non-sliding and non-targeted counterparts. Additionally, the authors demonstrated the stimuli responsiveness of the carriers by evaluating the release of doxorubicin, which was completely retarded at pH 7.4 (less than 1% in 48 h) and almost immediate at pH 5.5 (more than 90% in 1 h) [55].

**Co-delivery of different therapeutic agents for simultaneous targeting of several molecular pathways of the disease:** The resistance to chemotherapy remains a major challenge to effectively manage diseases such as leukemia. The combination of two or more drugs into one treatment protocol usually increases the therapeutic outcomes. This is achieved through modifications of specific cell signaling pathways with the aim to increase the cell sensitivity to at least one agent of the employed therapeutic drug combination. This approach is particularly attractive because single-drug therapeutic regimens are a rare commodity in chemotherapy [56]. The drug combination regimens offer the opportunity to target different molecular pathways that are unique for specific leukemia clones, thus overcoming possible drug resistance mechanisms and increasing the overall therapeutic effect. Still, it is challenging to coordinate pharmacokinetics, biodistribution, and intracellular concentration profiles of individual drugs with different physiochemical and biological properties [57–58]. Hence, current clinical combinatorial therapy regimens, which simply combine different drugs in a conventional dosage form, are far from a perfect chemotherapy approach for leukemia patients. It is logical to assume that loading multiple drugs onto a single nanoscale carrier will synchronize the corresponding therapeutic activities, thus maximizing the synergistic effect of the combinational chemotherapy regimen.

**Figure 3:**
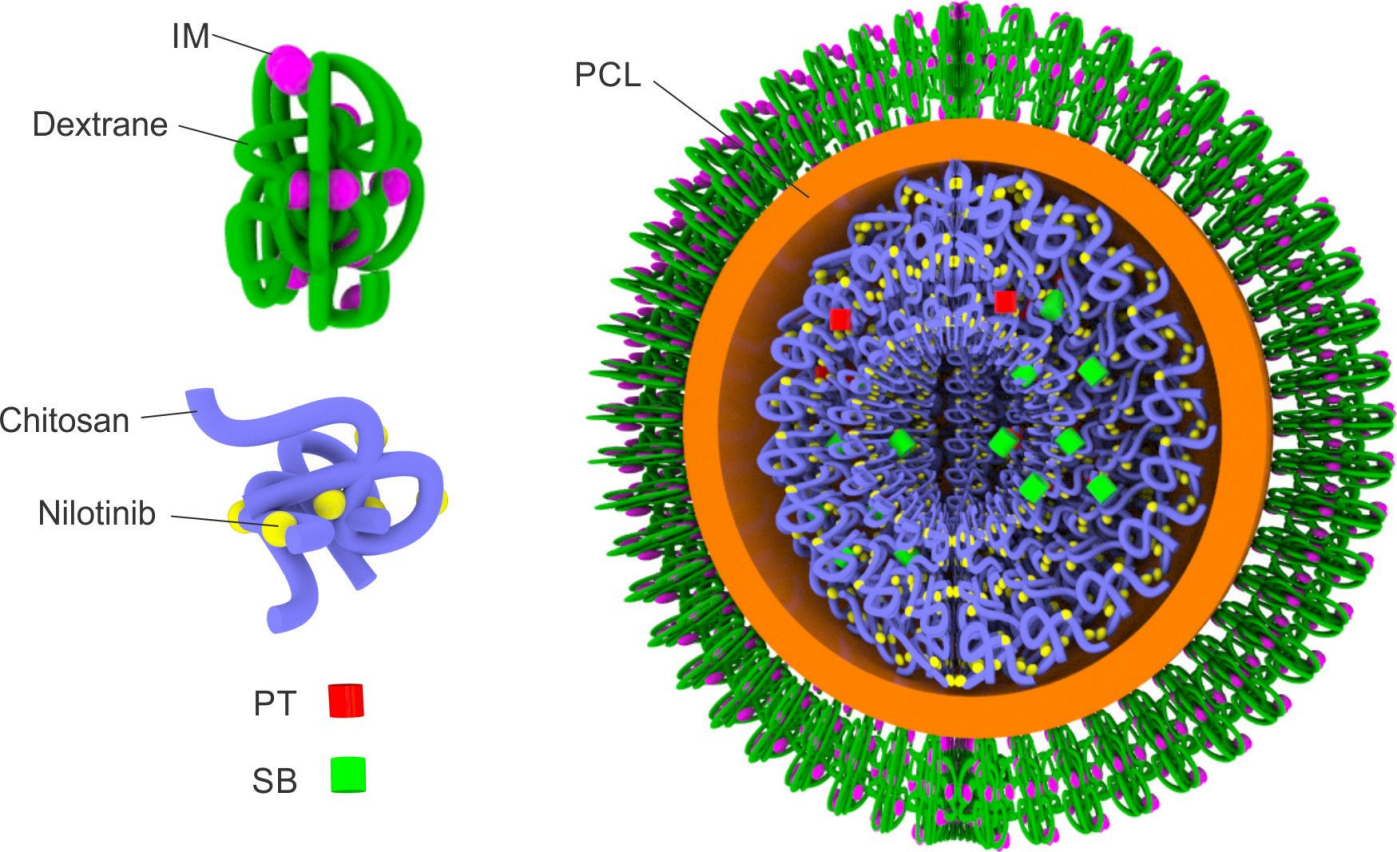
Wool-like hollow polycaprolactone (PCL) NDDS for the sequential co-delivery of imatinib mesylate (IM) and nilotinib (PT: potassium tartrate, SB: sodium bicarbonate). Nilotinib is released at low pH values as pores are formed in the PCL shell due to CO_2_ generated by PT and SB (preferably in the lysosomes), followed by IM release from the dextrane complexes in the cytoplasm due to the activity of the intracellular protease. Adapted from [62].

Clinical observations have revealed that co-administration of imatinib mesylate (IM) and other TKIs, such as nilotinib or dasatinib, may yield additive/synergistic anti-leukemia effects in CML [59–60]. Considering this, the development of a nanoscale targeting system for the co-delivery of these drugs appears to be a rational approach for combinational chemotherapy [61]. It has been shown that nilotinib, in the chemotherapeutic combination with IM, has a critical role in inhibiting or eradicating the several subclones that are resistant to IM. Cortese and co-workers developed wool-like hollow polymeric nanoparticles loaded with the abovementioned drug combination for the treatment of CML (Figure 3) [62]. The authors developed core–shell nanoparticles from polycaprolactone (PCL). The core of the nanoparticles was loaded with chitosan-complexed nilotinib, while the shell was loaded with a protease-sensitive dextrane–IM complex. In addition, the core was loaded with sodium bicarbonate and potassium tartrate, a mixture that quickly generates CO_2_ in an acidic environment and, thus, produces large pores in the nanoparticles, resulting in a burst release of the core contents. In this way, the authors managed to achieve a stimuli-responsive sequential drug release pattern. IM will be released in the cytoplasm from the dextrane complexes due to the activity of intracellular protease while the nilotinib–chitosan complexes will be released in the acidic environment of the lysosomal compartment following the generation of CO_2_. The first released IM should induce a partial inactivation of BCR-ABL oncoprotein, while the second released nilotinib should complete the oncoprotein inactivation, thus substantially reducing the probability of resistance. The authors reported that the combinatorial delivery of IM and nilotinib demonstrated a substantial reduction of the IC_50_ value regarding the KU812 CML cell line, compared to the free drugs. Also, it was noticed that the blockade of the G2/M phase played the main role in cell cycle arrest, demonstrating a sensitiveness of the cells to nilotinib that is attributable to IM.

Mendonca and co-workers worked on developing a targeted NDDS for а combination of siRNA and TKI in CML treatment [63]. The authors developed sterically stabilized liposomes decorated with transferrin for co-delivery of siRNA and IM for specifically silencing the BCR-ABL oncogene. In this case, IM enables the pre-sensitization of tumor cells, as a prerequisite for effective gene silencing, mediated by the siRNA. The results demonstrate that the siRNA/IM ratio affected the efficacy in BCR-ABL silencing and reflected on the evaluated IC_50_ values of IM on different cell lines. Additionally, the comparative analysis of the IC_50_ values on different cell lines revealed that the transferrin receptor expression and the cellular levels of BCR-ABL mRNA affected the efficacy of the formulation. The cell lines with higher levels of transferrin receptor expression and lower BCR-ABL mRNA levels were more sensitive to the prepared liposomal formulation. The importance of co-delivery of both agents was also observed as lower siRNA/imatinib ratios were needed to achieve IC_50_ values equivalent to those of the experiments in which the cells were exposed to a combination of encapsulated siRNA and free IM. This research highlights the need of achieving specific intracelullar concentrations and drug ratios in order to increase the efficacy of chemotherapeutic regimens, indicating the importance of co-delivery systems in the improvement of the overall therapeutic efficacy.

A targeted NDDS for the co-delivery of ponatinib and SAR302503 (selective JAK2 inhibitor) for efficient CML treatment was developed by Mu and co-workers [47]. The authors employed alendronate-decorated PLA–PEG micelles for BM targeting. Both ponatinib and SAR302503 are hydrophobic drugs and were co-encapsulated inside the hydrophobic core of PEG–PLA micelles by solvent displacement nanoprecipitation. The results demonstrated that the targeted NDDS enabled a high availability of the drugs inside the BM, which is a prerequisite for overcoming the dose-dependent side effects of both drugs. In part, this was also accomplished by the low-dose synergistic effect of countering different biological signaling pathways, with the goal of achieving short-term BCR–ABL1 kinase inhibition that resulted in induction of apoptosis in BCR–ABL1-positive and primary CML progenitor cells. The targeted NDDS significantly increased the survival rate of the murine leukemia models relative to the groups treated with single drugs, non-targeted co-delivery NDDS, and the untreated control. In addition, the low-administered dose of both co-delivered drugs and the NDDS itself proved to be non-toxic to healthy BALB/c mice.

The co-delivery strategy has the potential to overcome therapy-induced niche-mediated leukemia resistance in AML. Dong et al. developed a biomimetic NDDS composed of a mesoporous silica nanoparticulate core loaded with daunorubicin and coated with NALM-6 cell membrane vesicles that were further decorated with aTGFβRII antibodies, attached via hypoxia-sensitive azobenzene linker. The membrane coating plays a dual role in the formulation. It guides the drug to the BM via receptor–ligand interaction between CXCR4 (a chemokine receptor expressed on NALM-6 CM) and SDF-1 (a molecule of the BM endothelium) and mediates the hypoxia-driven release of the attached aTGFβRII antibodies in the BM, which then interfere with the interplay among LSCs and the BM niche cells. The remaining NALM-6-coated silica nanoparticles with daunorubicin are taken up by the leukemia cells, in which the drug is released, leading to high intracellular concentrations [64]. The results from this study highlight the importance of the sequential order of release of the therapeutic molecules in the co-delivery system and the formulation design approach to achieve such a complicated task. When one of the employed molecules interferes with the possible resistance mechanisms of the target cells, it is of prime importance to enable its release from the co-delivery system prior to the pharmaceutically active drug(s).

#### Future perspectives in the design of nanomedicines for targeting myeloid leukemia

Nanomedicine offers new perspectives and promising approaches in the therapy of myeloid leukemia. With the advances in the selective delivery of a variety of therapeutic molecules, from conventional drugs to proteins and nucleic acids, some of which were not available in the conventional treatment protocols due to unfavorable physicochemical and pharmacokinetic properties, nanotechnology offers strategies for targeting a variety of leukemia cell populations. Even though liquid tumors mainly reside in the blood circulation and seem readily available for chemotherapy, the subpopulation of leukemia stem cells that is mainly responsible for tumor progression and resistance is capable of evading treatment within the bone marrow microenvironment and to adapt through self-renewal, clonal expansion, and additional mutations. Therefore, LSC targeting is considered as one of the primary therapy goals in myeloid leukemia. Hence, the design of the nanoscale carriers needs to integrate strategies for extravasation in the bone marrow niches and subsequent specific molecular recognition of the LSC. Current advances in molecular diagnostics offer a glimpse into the molecular profile of each tumor subtype and define its common chromosomal translocations, shared mutations in oncogenes, gene expression profiles, and immune phenotypes. Such knowledge is important in the development of ligands for targeting a specific subpopulation of cells, based on their molecular profile and specific surface-exposed molecules. Considering that leukemia has shown a vast genomic heterogeneity, the integration of the molecular profile of the tumor into the design of the multimodal nanoscale carriers will have a tremendous impact for the future development of personalized nanomedicine for a safe and efficacious treatment of myeloid leukemia.

In addition, the co-delivery of different therapeutic molecules using nanomedicines is of great importance in the treatment of resistant cancer clone populations. Drug combinations for targeting different molecular pathways are available in conventional chemotherapy regimens. However, in order to achieve maximal therapeutic effects and to avoid drug resistance mechanisms, both drugs need to be released inside the cells simultaneously or in a specific sequence. This can be accomplished by formulating combined drug therapy in a nanoscale carrier. This treatment modality highlights the importance of NDDSs in the safety and efficacy of future chemotherapeutic regimens for myeloid leukemia.

### Molecular profiles of solid tumors – the role of EGFR and CD44

Overexpression of epidermal growth factor receptor (EGFR) or its altered activity due to mutations is a common trait in many cancers [65]. It is a compelling marker of many malignances significantly associated with cancer progression, metastasis, and drug resistance. Also, EGFR is a promising molecule for targeted therapy. Different drugs against EGFR have been introduced in the clinical practice [66]. However, the efficacy of currently developed agents used for blocking EGFR signaling pathways, such as TKIs and monoclonal antibodies (mAbs) preventing EGFR to be activated by its ligands, is constantly challenged by innate and acquired resistance. Therapeutic inefficacy may be overcome by the design of nanoscale systems, based on receptor-mediated endocytosis, that carry several functional agents against multiple intracellular pathways or different components of the EGFR signaling network. The systems are (i) nanoscale systems capable of downregulation or inhibition of positively correlated receptors in order to improve the sensitivity of the cancer cells against the antineoplastic agents, (ii) functionalized nanoscale systems carrying anticancer agents that target multiple receptors, or (iii) other multifunctional approaches involving gene therapy for receptor knockdown administered with anticancer agents in targeted nanomedicines [67]. Cluster of differentiation 44 (CD44) promotes carcinogenesis and progression. It also acts as a co-receptor in the EGFR signaling cascade [2,68]. The expression level of CD44 is positively correlated with the wild-type EGFR level in cancer tissues. Its downregulation/inhibition significantly accelerates the degradation of EGFR. This increases the sensitivity of the cancer cells to antineoplastic agents and contributes to overcome multidrug resistance. Overwhelming evidence of mechanisms involving different receptors, signaling networks, and ligands collaborating together in cancer development and survival have fostered the parallel development of multifunctional bioactive nanomedicines capable of attacking multiple targets, which hold potential to overcome current deficiencies of targeted therapy.

#### Targeting strategies with innovative ligands for nanoparticle surface engineering

**Targeted delivery of anticancer agents using nanoparticles decorated with EGFR ligands:** Molecular profiling of solid tumors is already a valuable tool for molecular classification, outcome prognosis, and the prediction of therapy, efficacy, and toxicity of current chemotherapy regimens. The screening of individual cancer signatures is an increasingly important aspect in the design of novel targeted therapies and nanoscale carriers for personalized cancer treatment. Precision in the targeting of nanoscale carriers to specific cancers or cancer populations is gaining momentum ever since the development of simple techniques for the prediction and synthesis of native ligands for specific receptors. Advances in molecular targets for cancer therapy and their implications in nanoscale targeting strategies will be briefly presented in the following part of the article.

EGFR is a member of the ErbB family of receptors, a subfamily of closely related receptor tyrosine kinases, which includes ErbB1 (also known as EGFR), ErbB2 (HER2/neu), ErbB3 (Her 3), and ErbB4 (Her 4). Two types of pathological alterations of EGFR in cancers, the kinase-activating mutation in EGFR and the overexpression of the EGFR protein, are common traits in many solid tumors and validated delivery targets for several cancers including lung, colorectal, and certain subtypes of breast cancer [69]. For instance, overexpression of EGFR and DNA mutations in the extracellular and intracellular portions of the protein have been observed in 43–89% of cases of non-small-cell lung carcinoma (NSCLC) [70], which makes this receptor a relevant target in NSCLC treatment. Two distinct therapeutic approaches developed for targeting EGFR in various human malignancies are the use of mAbs (binding to extracellular domains) and tyrosine kinase inhibitors (targeting the intracellular TK domain). In addition to the monoclonal antibodies, different EGFR-specific targeting ligands were developed for the extracellular EGFR domain, such as single-chain antibody fragments, antibodies, recombinant epidermal growth factor (EGF), and EGF peptide mimetics.

At the site of action, mAbs against EGFR competitively inhibit ligands, promote receptor internalization, and prolong downregulation induction. Antibody-dependent cell-mediated cytotoxicity and, to a lesser extent, complement-mediated cytotoxicity are the mechanisms of the therapeutic action of mAbs [71]. Used as specific motifs for the surface decoration of nanoscale carriers, mAbs generally improve the capacity of receptor-mediated active endocytosis and enhance the intracellular delivery of the carrier cargo [72–73]. However, it is ambiguous whether the downregulation effect is preserved when mAbs are used as ligands for targeting diagnostic or therapeutic nanoparticles. Qian et al. used cetuximab (C225 mAbs), an EGFR-neutralizing monoclonal antibody for cancer treatment, as a targeting ligand for AuNPs [74]. The authors evaluated the efficacy of C225 mAbs to target EGFR and improve the internalization, the chemical sensitivity of the cancer cells, and the efficacy of the gold nanoparticles, using different types of EGFR-expressing NSCLC cancer cell lines. C225-AuNPs showed the largest inhibitory effect on cell growth and cell proliferation when the NSCLC cell line A549 with high EGFR expression was used. In addition, it was pointed out that the cell proliferation was inhibited due to the significantly increased rate of apoptosis in the A549 cell line, while no alteration of the cell cycle distribution was noticed. The effects in the H1299 cells with low EGFR expression were negligible. The authors hypothesized that the interaction between EGFR and C225-AuNPs influenced cell internalization, EGFR signaling, and downstream protein levels. Their results showed that proliferation-related p-Akt and p-Erk levels were both significantly downregulated, and apoptosis-related Bcl-2 levels were upregulated after treatment with C225-AuNPs, compared with the treatment with C225 or IgG-AuNPs, in cell lines with high EGFR expression [74]. All things considered, EGFR ligands such as C225 may play a dual role. They may actively target the nanosystem and enhance cell internalization, while at the same time activating the extracellular domain of membranous EGFR and influencing the EGFR signaling pathways through increased endocytosis (receptor internalization) and cytoplasmic accumulation of EGFR.

In the study of Yokoyama et al. paramagnetic gold-coated plasmonic NPs (40–50 nm) with iron core and anti-EGFR antibody (clone 225) functionalization were designed. Their efficacy against human NSCLC cells was evaluated. Increased antitumor efficacy of C225-NPs by inducing apoptosis and autophagy, compared to C225 and the carrier, was reported. The authors mentioned the importance of the three-dimensional arrangement of macromolecular biopharmaceuticals, especially of nanoscale templates, for the interaction of the nanoscale systems with cancer cells. Attaching C225 to NPs was important for the enhanced tumor cell killing. A mixture of free C225 and NPs did not exhibit the same degree of cell killing activity. Also, in contrast to C225-NPs, free C225 antibody did not induce autophagy in cells [75]. Maya et al. reported the design and evaluation of EGFR-targeted cetuximab–chitosan cross-linked γ-poly(glutamic acid) nanoparticles loaded with docetaxel. The NPs were prepared by cross-linking the NH_2_ groups of chitosan with the carboxylic groups of poly(glutamic acid). EDC/NHS chemistry was used for conjugating the mAbs on the surface of the nanoscale carriers. The cross-linked nanoparticles showed superior antiproliferative activity compared to NPs without ligands. In vitro cell culture studies on A549 cells pointed to increased apoptotic and necrotic cancer cell death after treatment with nanoscale carriers with specific EGFR targeting motifs, most probably due to improved uptake of docetaxel-loaded cross-linked nanoparticles and the EGFR receptor internalization [76]. There is a plethora of research articles regarding the use of EGFR mAbs as a specific targeting motif for polymer NPs such as poly(lactic acid-*co*-lysine), poly(ethylene glycol-*co*-caprolactone), an poly(lactic acid-*co*-glycolic acid) NPs. All of them have shown improved tumor targeting or internalization, as well as enhanced efficacy in vitro or in vivo [77–81].

Nonetheless, when combining a ligand with a nanoscale carrier, a number of factors that may influence the targeting efficacy as well as the efficacy and safety of the drug delivery system has to be considered. The most effective ligand has to be selected, which is complicated by the lack of standard methods for the evaluation of the targeting specificity, safety, and efficacy of NPs. In general, the large size and the limited number of mAbs that might be anchored at the NP surface, their immunogenicity, the risk of activation of compensatory mitogenic signaling pathways, and the engagement of growth-promoting cues that compensate for inhibition of the targeted kinase, may reduce the efficacy and safety of mAbs and their use as a targeting ligands [82–83]. Further, the native EGF ligand, although much smaller than mAbs, will influence cell proliferation and survival through downstream signaling cascades. It may, therefore, activate compensatory signaling and amplify cancer growth [84]. Today, there are various approaches of actively targeting EGFR besides using the native ligand or mAbs to enhance the delivery of therapeutic and diagnostic agents to EGFR-overexpressing cancers. The ligands include antibody fragments, functional oligonucleotides developed by combinatorial methodologies, aptamers, and EGFR-specific peptides of low molecular weight [85].

**Figure 4:**
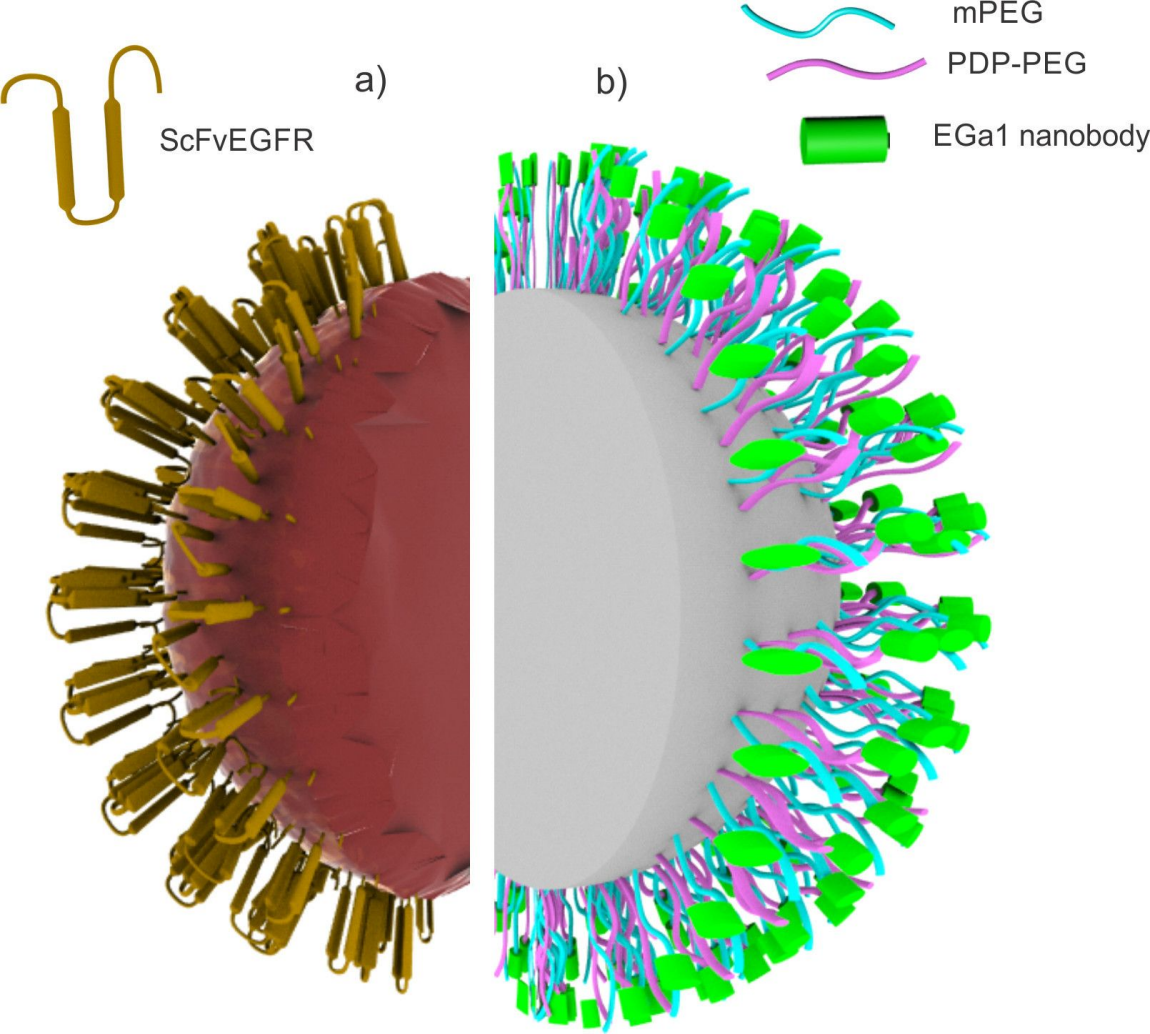
EGFR targeting strategies using “non-conventional” ligands. (a) Heparin nanoparticles with surface-oriented recombinant single-chain antibody (ScFvEGFR) for efficiently targeting EGFR-positive NSCLC cells; adapted from [81]. (b) (mPEG/PDP-PEG)-*b*-p(HPMAm-Lacn) nanomicelles decorated with EGa1 nanobodies for selectively targeting EGFR-positive A431 and UM-SCC-14C cells; adapted from [98].

Commonly used are single-chain recombinant antibodies against EGFR (ScFvEGFR, *M*_W_ = 25 kDa), which contain the specific EGFR binding region but lack the Fc region. In order to increase their functional affinity, single-chain antibodies are converted into a multivalent form, which allows for targeting two antigens simultaneously and for additional functionalities such as effector functions, cytotoxic cells recruitment, or the delivery of immunodiagnostics. Further, the binding affinity can be controlled in order to optimize tumor penetration and selectivity for targeting [86]. Peng et al. developed ScFvEGFR–heparin nanoparticles for the targeted delivery of cisplatin to EGFR-positive NSCLC cells (Figure 4a). The antitumor activity was significantly enhanced without weight loss or damage to kidney and spleen in nude mice bearing EGFR-expressing non-small cell lung carcinoma [81].

Variable heavy homodimers (VHH), that is heavy-chain-only antibody fragments or nanobodies, are more stable, smaller, and exhibit increased solubility and specificity. They are non-immunogenic, antigen-binding fragments with considerable therapeutic activity and huge potential for homing of drug-loaded nanoscale carriers and diagnostic agents to tumors [87]. Compared to monoclonal antibodies, which contain two heavy and two light chains, nanobodies are variable fragments of heavy-chain-only antibodies, which are considered to be non-immunogenic due to the high similarity with human VH sequences [88]. These innovative targeted therapeutic tools, produced by cloning the variable domain of heavy-chain antibodies, combine high stability and solubility, low immunogenicity and excellent affinity and specificity against all possible targets including tumor markers. Oligoclonal nanobodies, which recognize different epitopes at the same antigen, or multivalent nanobodies can be targeted against extracellular targets for therapeutic purposes in order to inhibit receptor–ligand binding or receptor activity in vitro and in vivo. Used as specific motifs at the surface of different nanoscale carriers, nanobodies may contribute to improved tumor targeting and cell internalization [89–91]. Literature data for different types of polymer nanoscale carriers decorated with anti-EGFR nanobodies points to an improved delivery of the NPs and the cargo to the EGFR-overexpressing cancer cells through EGFR-mediated internalization, and to an enhanced therapeutic efficacy due to the antagonistic activity [92–97].

Talelli et al. synthesized nanobody-conjugated PDP-PEG-*b*-p(HPMAm-Lac*_n_*) micelles for the treatment of EGFR-overexpressing cancers (Figure 4b). The thermosensitive diblock copolymer ω-methoxy-poly(ethylene glycol)-*b*-poly[*N*-(2-hydroxypropyl)methacrylamide lactate] (mPEG-*b*-p(HPMAm-Lac*_n_*) and a pyridyldithiopropionate (PDP)-functionalized (mPEG-*b*-p(HPMAm-Lac*_n_*) diblock copolymer (PDP-PEG-*b*-p(HPMAm-Lac*_n_*)) were synthesized and used for micelle preparation. Further, EGa1 nanobody was coupled to the surface of the (mPEG/PDP-PEG)-*b*-p(HPMAm-Lac*_n_*) nanomicelles in order to enhance the specificity of the nanoscale carriers to EGFR-positive cancer cells and to improve recognition and intracellular uptake. Compared to micelles without ligands and to EGFR-negative cell lines, a significantly increased binding and internalization in EGFR-overexpressing cells was noticed, which confirmed the interaction of the nanobody at the micelle surface with EGFR [98].

During the last decade, the phage display process helped in the high-throughput screening of protein interactions, in the discovery of peptides and proteins for biosensors, in the determination of tumor antigens, and in the invention of new and more efficient drugs and improved mechanisms of drug delivery. Peptide ligands, developed by screening phage display libraries are promising targeting moieties for the selective delivery of radionuclides, anticancer drugs, and therapeutic genes to tumors. They benefit from a small size, unique conjugation possibilities to various drugs, vectors, or nanoscale carriers, enhanced intra-tumoral diffusion, high specificity, simple and affordable production, non-immunogenicity, and low toxicity [99]. Recently, three targeting phages (HPC1, HPC2, and HPC4) and the corresponding displayed peptides (HSP1, HSP2, and HSP4) were identified, using phage display biopanning of H460 lung cancer cells, to be specific to small-cell lung carcinoma (SCLC) and NSCLC cell lines, and to clinical specimens, but not to normal lung tissue. By using in vivo optical imaging of phage homing and magnetic resonance imaging of peptide-SPIONs it has been shown that HSP1 was tightly bound to cancer cell surfaces, in contrast to HSP4 peptide, which was preferentially endocytosed. Therefore, HSP4 peptide may be used for improving targeting as well as for an enhancement of cell internalization of different nanoscale carriers and theragnostic devices in lung cancer. Liposomal doxorubicin conjugated to HSP1, HSP2, and HSP4 showed significantly greater therapeutic efficacy than non-targeted liposomal drugs in NSCLC (H460 and H1993) animal models [100].

In the light of these discoveries, novel targeting peptides as alternative EGFR-binding ligands for the delivery of diagnostic agents and/or anticancer drugs to solid tumors were also developed. An enriched phage clone encoding the amino acid sequence YHWYGYTPQNVI, designated as GE11 ligand, with speciﬁc binding capabilities to EGFR was developed using phage display peptide library screening. Peptide ligands, in contrast to native EGF ligands, are readily diffusible and show very low mitogenic and neoangiogenic potential and low immunogenicity. They can be easily incorporated in gene delivery vectors as a selective targeting moiety for targeting reporter genes to the EGFR-overexpressing lung cancer cells in vitro and in vivo [83].

The peptide D4 is another example of an EGFR peptide ligand capable to bind to a surface pocket of EGFR. A liposome with a D4-modified surface has shown high affinity to EGFR receptor and enhanced endocytosis in a human NSCLC cell line derived from a lymph node. Improved targeting in vivo in H1299 xenograft tumor tissues was also reported by the authors [101].

The structure of small peptides for targeting EGFR-active cancers may also be predicted by computer-assisted design (CAD). Recent research data has proven computer-assisted techniques as extremely promising. A combinatorial approach of screening of phage display libraries with CAD as well as hydropathic analysis, and comparative sequence/structure analysis, may facilitate the identification and synthesis of small peptide ligands that mediate internalization and down-regulation of EGFR. It has been shown in the literature that the simultaneous autophosphorylation of the three carboxyl-terminal tyrosine residues (Y1068, Y1148, Y1173, the three major autophosphorylation sites of EGFR (EGFRAPS)) is important for rapid internalization and degradation of the EGF receptor [102–103]. Therefore, the prediction of the structure of binding peptides with high affinity for the EGFRAPS might be a very promising approach for the design of therapeutic agents or ligands for the treatment of EGFR-overexpressing cancers [104]. With the knowledge of the contact sites between the natural ligands and the EGFRAPS assembly and the crystal structure of their complexes, the design of small peptides for EGFRAPS can be aided by computer analyses. Han et al. evaluated the binding activities of small peptides of the EGFR C-terminus, using FITC labeling and flow cytometry for measuring the binding rates, the internalization rates of peptides, and the overall efficacy of the peptide ligand. They used the LARLLT protein, which binds to putative EGFR selected from a virtual peptide library by computer-aided design, as a positive control and the independent peptide RALEL as a negative control. Their experimental results indicated that the AEYLR peptide ligand could specifically bind to human EGFR and human non-small-cell lung tumors that express EGFR. Conjugating AEYLR as a targeting element to different molecules pointed to its ability to deliver the cargo (anticancer agent, radiotherapy, or gene therapy) directly to the cell/cell nuclei. A synergistic effect and increased therapeutic efficacy was also noticed due to the inhibition of the receptor and suppression of autophosphorylation of EGFR. Further experiments showed that AEYLR, derived from the C-terminal of EGFR, did not stimulate cell proliferation and is safe for use as a targeting agent [84].

#### Simultaneous targeting of several molecular pathways using multifunctional NPs

**Figure 5:**
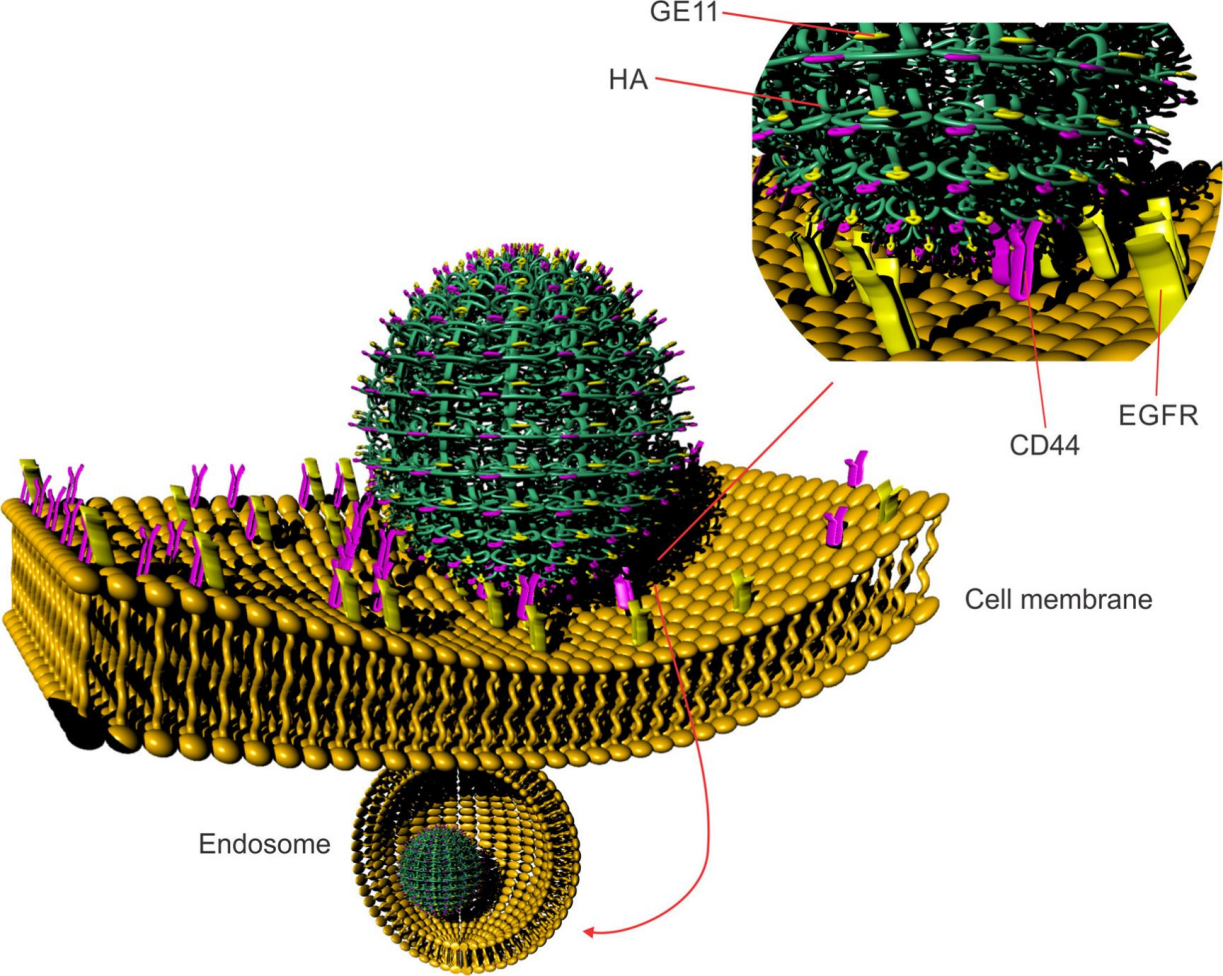
A multifunctional hyaluronan nanogel targeting EGFR and CD44. The binding of hyaluronan residues and GE11 peptide to CD44 and EGFR, respectively, triggers receptor-mediated endocytosis of the nanoscale carrier. Adapted from [108].

Cluster determinant 44 (CD44) is an important signaling platform that integrates microenvironmental signals with growth factor and cytokine signaling. CD44 can promote uncontrolled growth, evasion of apoptosis, angiogenesis, cell motility, and invasion, either independently or in collaboration with other cell-surface receptors [105]. Several studies have reported that CD44, and especially the CD44 splice isoform CD44s, positively correlates with EGFR tumor signatures and predicts poor survival in different types of cancers [68,106]. Literature results support the existence of a CD44–EGFR axis in colon, liver, lung, and pancreatic cancers, and the role of CD44s in EGFR signaling. In fact, by interacting with the small GTPase Rab7A, CD44s inhibits Rab7A-mediated EGFR trafficking to lysosomes and the subsequent degradation of EGFR [107]. The role of the EGFR–CD44 axis in cancer progression might be the reason behind the limited success of targeting only EGFR as a therapeutic approach in cancer treatment. For example, a high EGFR expression is very common in glioblastoma multiforme (GBM) but its main feature is the limited efficacy of EGFR inhibitors. Wang et al. showed that an experimental approach comprising CD44 depletion combined with EGFR inhibition resulted in a synergistic killing of GBM cancer cells [107]. Recently, Xu et al. pointed out to an EGFR pathway for CD44 upregulation and its robust impact on the development of breast cancer. They noted that CD44 acted downstream of EGFR in the progression of breast tumor. There is no doubt that a deeper understanding of the interaction between CD44 and EGFR in cancer progression will provide better approaches to cancer treatment, and that combined EGFR/CD44 targeting may be the future direction in the treatment of some types of cancer [106]. Chen et al. published results on Granzyme B (GrB)-loaded multifunctional hyaluronan nanogels targeting EGFR and CD44 (EGFR/CD44-NGs) (Figure 5). Their flow cytometry assay results pointed to a sixfold improved uptake of the multifunctional nanogel, in CD44- and EGFR-positive SKOV-3 ovarian cancer cells, compared to CD44-NG, as well as to increased cell apoptosis in vitro. GrB-EGFR/CD44-NGs induced nearly complete growth suppression of the tumor in xenografted tumor models in nude mice [108].

Suda et al. published interesting experimental results for CD44 overexpression in relation to an epithelial-to-mesenchymal phenotype transition (EMT) and the acquired resistance to EGFR TKIs in lung cancers. With a contribution of CD44, different biochemical changes in cells lead to the transition of polarized epithelial cells to mesenchymal cells characterized by enhanced migratory capacity, invasiveness, elevated resistance to apoptosis, and greatly increased production of extracellular matrix (ECM) components. After completion of the EMT and degradation of the underlying basement membrane, mesenchymal cells can migrate away from the epithelial layer, contributing to the aggressiveness of the tumor. The authors have pointed out that shRNA-mediated knockdown of CD44 reversed the EMT [109]. Further, there is ample evidence that CD44 is a cancer stem cell marker and that its overexpression enhances the invasion of cancer stem cells (CSCs). CSCs are distinguished through biomarker combinations, such as CD44+/CD24 for prostate cancer stem cells and triple-negative breast cancer stem cells [110]. Especially, CD44v isoforms are frequently found as critical cancer stem cell markers for regulating the properties of CSCs, including self-renewal, tumor initiation, metastasis, and chemoradioresistance [111]. However, the poor understanding of the involvement of different CD44 isoforms, such as CD44/CD44v and CD44/CD44s, in signaling modulation may have an impact on their use in clinical applications. Additionally, the contribution of hyaluronan (HA), a major component of the extracellular matrix, in providing a favorable environment for tumors and especially for stem cell renewal and maintenance, has been established recently. Steady-state levels of HA are generally quite low in most normal tissues; however, its levels dramatically increase in tumor tissues. HA has been associated with a poor prognosis of many different cancer types including lung, ovarian, bladder, and breast cancer. There is increasing evidence for the role of HA in the creation of a specific microenvironment that is favorable for tumor angiogenesis, invasion, and metastasis. Moreover, it is well known today that excessive HA production promotes the acquisition of CSC signatures through EMT [112–113]. Schwertfeger et al. interpreted the interplay among the tumor, its stromal microenvironment, and the pro-tumorigenic inflammatory environment using breast cancer as a model [114]. A pro-inflammatory environment is usually evoked by the interaction of low-molecular-weight hyaluronan (LMW-HA) with HA receptors (the fragmentation of high-molecular-weight HA (HMW-HA) is promoted by free oxygen or nitrogen radicals and the release of hyaluronidases within the tumor microenvironment). HA has a dual function in the tumor microenvironment. In breast cancer, LMW-HA also induces recruitment and activation of inflammatory macrophages, which will further release NF-κB-regulated pro-inflammatory factors normally involved in tissue repair. This inflammation-supporting machinery from the wound healing process is immediately used by the tumor for fast growing and development. Additionally, HA modulates the function of tumor-associated macrophages to support CSCs, and evokes a wide range of signals required for CSC self-renewal through intensive HA–CD44 interactions. Activated HA–CD44 signaling through various receptors including CD44, CD168/RHAMM, and toll-like receptor 4, regulates migration, invasion, adhesion, survival and cell differentiation, EMT, and metastasis. The latter is regulated in collaboration with the tyrosine kinase (TK) and WNT/β-catenin signaling systems and receptors of these signaling families, such as VEGFR, EGFR, c-Met, and LRP6 cellular receptors. In malignant breast carcinoma cells, the HA–CD44 interaction activates multiple TKs, as well as the assembly of lipid-raft-integrated signaling complexes, which strongly promote apoptosis resistance in cancer cells. Targeting the HA–CD44 communication might be an efficacious and rational approach to fight against malignant breast carcinoma. In addition, there is ample evidence to prove that a blockade of the HA–CD44 interaction causes the disassembly of macromolecular lipid-raft-integrated complexes and the inactivation of TKs in breast, lung, colon, and prostate cancer cells [115–119]. Recent findings from The Cancer Genome Atlas (TCGA) and the International Cancer Genome Consortium (ICGC) verified that although each cancer seems to be unique in its genetic mutations, there is a range of signaling pathways that are frequently affected within particular cancer types [120]. Further, the HA–CD44 interaction regulates drug transporter expression and augments MDR1 expression, increasing drug resistance in cancer cells. Moreover, MDR1 is associated with CD44 in lipid microdomains of the cell membrane, and MDR1 gene expression can be stabilized by CD44 [121–122]. It is obvious that targeting the HA–CD44 interaction can inhibit tumor survival and renewal processes at multiple stages in various types of cancers [112–113]. Therefore, a better understanding of the alteration of HA homeostasis in the tumor microenvironment and the molecular mechanisms involved in controlling HA and CD44 during cancer progression may significantly contribute to increase the efficiency of anticancer therapies.

HA indirectly affects cellular function through its activity in the assembly and remodeling of the ECM. It also regulates different cellular processes, mainly through CD44 and RHAMM signaling, although it has been shown to affect toll-like receptor signaling as well. RHAMM, that is, receptor for hyaluronan-mediated motility, has been shown to have extracellular functions (i.e., it coordinates HA-induced cell growth and motility with other cell surface receptors in lung cancer) and intracellular functions (i.e., it modulates cytoskeletal organization through interaction with microtubules and actin filaments and contributes to ERK activation in breast cancer) [123]. Also, variant forms of RHAMM are found on cell surfaces and inside cells [124]. Different studies indicate coordinated actions between CD44 and RHAMM and the necessity of RHAMM surface expression during CD44-mediated cell migration in tumorigenesis and inflammation. However, the mechanisms of cooperativity between RHAMM and CD44 as well as the interaction of HA with these receptors in the processes of tumor cell migration and adhesion are not fully explained.

Clinical relevance of RHAMM expression in NSCLC attracts a lot of research interest. The expression of RHAMM mRNA is, respectively, twelve- and tenfold higher in lung adenocarcinoma and squamous lung carcinoma than in the corresponding healthy lung tissues [125]. Recently, Wang et al. found that RHAMM mRNA expression correlated with stages of differentiation and inferior survival in more than 400 cases of lung adenocarcinoma in the Director's Challenge cohort. They indicated that out of four RHAMM splice variants, RHAMMv3 (also known as RHAMMB) is the dominant variant in NSCLC. The authors also showed that successful shRNA-mediated knockdown of RHAMM reduced the migratory ability of H1975 and H3255 lung adenocarcinoma cell lines [123]. Considering that, according to literature data, there is no more than 20% molecular alterations and rearrangements of the routine biomarkers, such as EGFR and anaplastic lymphoma kinase (ALK) [123], in lung adenocarcinoma. A more rational approach to determine predictive biomarkers and therapeutic targets in NSCLC would be to focus on CD44, RHAMM, and their communication with HA.

Advanced research in cancer genomics and their mutational repository have fostered parallel developments in the field of bioactive nanomedicines designed with abilities to attack specific targets at the cancer cell surface, inside the cells, or at the level of the ECM (Table 2). Self-assembled block copolymers such as (i) PEG-*b*-PAA, (ii) PEG-*b*-polyester block copolymers ((mPEG-*b*-poly(ᴅ,ʟ-lactide), PEG-*b*-PLA, and PEG-*b*-PCL), (iii) triblock PEG-polyester block copolymers (PCL-*b*-PEG-*b*-PCL, PLGA-*b*-PEG-*b*-PLGA, PEG-*b*-PCL-*b*-PEG, and PEG-*b*-PLGA-*b*-PEG), and (iv) PEG-polypeptide polymers (PEG-poly(glutamic acid) (PGlu), PEG-poly(ʟ-lysine) (PLL), and PEG-poly(aspartic acid) (PAsp)), and PEG-conjugated lipids [126–127] are re-evaluated for their targeting efficacy and decorated with novel targeting ligands. Natural polymers such as heparin, chitosan, dextran, and other polysaccharides, their derivatives and conjugates, as well as hyaluronan are also attracting a lot of interest. Among the naturally occurring polysaccharides, hyaluronan has been extensively investigated regarding HA-binding receptors, such as the CD44 receptor, the RHAMM, or the lymphatic vessel endothelial receptor 1 (LYVER-1), in cancer cells. HA is an anionic, linear glycosaminoglycan composed of alternating disaccharide units of ᴅ-glucuronic acid and *N*-acetyl-ᴅ-glucosamine with β(1,4) and β(1,3) glycosidic linkages [128]. It is readily degraded by hyaluronidases in the cytosol of tumor cells [129]. There are different examples for the application of HA in the design of targeted drug delivery systems. Within the last three years, around 50 research articles have been published describing hyaluronan-decorated gene- or drug-loaded nanomedicines in the form of polymers, lipid NPs or self-assembled systems, polymerosomes, or superparamagnetic iron oxide NPs for the diagnosis and treatment of breast, lung, colon, head, and neck cancer (according to Scopus). The chemical properties of HA and the fact that chemical modifications can be performed on three available functional sites of HA, (i.e., carboxylic, hydroxyl, and acetamido groups) for conjugation and cross-linking, have led to the development of drug–HA conjugates and surface-decorated nanoparticles for improved interaction with CD44-overexpressing cells or improved CD44-mediated uptake. Some of these systems have already entered clinical trials. Results from clinical trials with a paclitaxel–HA conjugate for the treatment of papillary non-muscle-invasive bladder cancer (NMIBC) were recently published by Bassi and co-workers [130]. The paclitaxel–HA conjugate used in this study was synthesized by activation of the hydroxy group of paclitaxel with carbodiimide for conjugation with 4-bromobutyric acid to form ester-linked 4-bromobutyric paclitaxel, which was conjugated to the carboxylic group of HA. The mechanism of action of paclitaxel–HA conjugates was evaluated using RT-4 and RT-112/84 urothelial carcinoma cell lines by Montagner and co-workers. They found that the conjugate is internalized by an energy-dependent mechanism that involves CD44. Upon internalization, the hyaluronan moiety is cleaved in the lysosomes, leading to cytoplasmic diffusion of the free drug, caspase activation, and disruption of the β-tubulin microtubular mesh with subsequent apoptosis [131]. Further, Bassi et al., in their clinical study, aimed to assess the ablative activity of intravesical administration of a paclitaxel–HA solution (Oncofid-P-B 600 mg) as well as the time to relapse after instillation. Complete response in patients with NMIBC was observed in 45% of the patients treated with six weekly intravesical administrations. A higher rate of complete response was reported in patients not previously treated with chemo/immunotherapy. The probability to be free of recurrence after 3, 6, 9, and 12 months from the first drug administration was 98.2%, 96.4%, 74.4%, and 58.9%, respectively [130].

**Table 2:** NDDSs in molecular targeting of solid tumors sorted by targeting ligands.

targeting ligand	NP type	payload	target	efficacy assessment	ref.

folic acid (FA)	NPs with poly(*N*-isopropylacrylamide)–carboxymethyl chitosan shell and PLGA core	NU7441 (potent radiosensitizer) and gemcitabine	FA receptor α	in vitro, in vivo	[132]
	solid lipid nanoparticles (SLNs) coated with a folate-grafted copolymer of PEG and chitosan	paclitaxel	FA receptor	in vitro	[133]
	polyethylene glycol-hydrophobically modified dextran (PEG-HMD) micelles	paclitaxel	FA receptor	in vitro, in vivo	[134]
	PLGA–PEG NPs	docetaxel and curcumin	FA receptor	in vitro	[135]
	gold NPs stabilized by quaternized chitosan–gallic acid–folic acid (Au@QCSGA-FA)	3,4,5-tribenzyloxybenzoic acid (GAOBn)	FA receptor	in vitro	[136]
	chitosan–folate conjugated multiwalled carbon nanotubes	docetaxel/ coumarin-6	FA receptor	in vitro	[137]

carbohydrates					

hyaluronan	NPs with chitosan shell and calcium phosphate core	cisplatin	CD44 receptor	in vitro	[138]
galactose	SLNs	doxorubicin	lectin receptors	in vitro	[139]
mannose	PLGA/histidine-based pH-responsive nanomicelles	gefitinib	lectin receptors	in vitro, in vivo	[140]

proteins					

transferrin (Tf)	liposomes	doxorubicin	Tf receptor, CD71	in vitro	[141]
	lipid-coated PLGA NPs	doxorubicin	Tf receptor	in vitro, in vivo	[142]
	PEG-modified chitosan NPs	paclitaxel	Tf receptor	in vitro	[143]
	SLNs	etoposide	Tf receptor	in vitro, in vivo	[144]
EGF	gelatin NPs	doxorubicin	EGFR	in vitro, in vivo	[145]
	gelatin NPs	cisplatin	EGFR	in vitro, in vivo	[146]

peptides					

LHRH peptide	mesoporous silica NPs	doxorubicin or cisplatin, and two types of siRNA	LHRH receptors	in vitro	[147]
synthetic analogue of LHRH	nanostructured lipid carriers	doxorubicin or paclitaxel, and two types of siRNA	LHRH receptors	in vitro, in vivo	[148]
CVKTPAQSC peptide	PLA NPs	docetaxel	—/	in vitro, in vivo	[149]
CSNIDARAC peptide	PEGylated liposomes	doxorubicin	—	in vivo	[150]
GE11 peptide	PEGylated liposomes	doxorubicin	EGFR	in vitro, in vivo	[151]
SP5-2	PEGylated liposomes	doxorubicin or vinorelbine	tyrosine kinase receptors, VEGFRI (Flt-1)	in vivo	[152–153]
iRGD	pluronic P85–polyethyleneimine/TPGS complex NPs	paclitaxel and survivin shRNA	integrin αvβ3 and neoropilin 1	in vitro, in vivo	[154]
LFC131 peptide	*O*-carboxymethyl chitosan nanoparticles	docetaxel	chemokine receptor CXCR4	in vitro	[155]
	sodium carboxylmethyl cellulose coated PLGA NPs	doxorubicin	chemokine receptor CXCR4	in vitro	[156]
AHSGMYP peptide	PLA NPs	docetaxel	—	in vitro, in vivo	[157]
TH10 peptide (TAASGVRSMH)	blended NPs composed of aldehyde-PEG–PLA and mPEG–PLA	docetaxel	NG2 proteoglycan	in vivo	[158]

aptamers					

EpCAM-fluoropyrimidine RNA aptamer	PLGA-*b*-PEG nanopolymersomes	doxorubicin	epithelial cell-adhesion molecules (EpCAMs)	in vitro, in vivo	[159]
AS1411 aptamer	PEG–PLGA nanopolymersome	gemcitabine	nucleolin	in vitro	[160]
	PLL–alkyl-PEI NPs	shRNA plasmid	nucleolin	in vitro	[161]
RNA aptamer	PLGA NPs	gefitinib	Ets1 (proto-oncoprotein)	in vitro, in vivo	[162]

Abs and fragments					

Fab* fragments of a monoclonal antibody	PEGylated liposomes	doxorubicin	β_1_ integrins	in vitro, in vivo	[163]
human single-chain variable fragment antibodies	PEGylated liposomes	doxorubicin	c-Met protein (receptor for hepatocyte growth factor)	in vitro, in vivo	[164]
cetuximab	PLGA NPs	paclitaxel palmitate	EGFR	in vitro, in vivo	[165]
	PLA NPs	gemcitabine	EGFR	in vitro	[166]
IgG, mAb 174H.64	PEGylated liposomes	doxorubicin	cytokeratin-associated antigen [167] expressed in mammalian squamous carcinoma	in vitro, in vivo	[168]

HA-decorated environmentally responsive nanomedicines are particularly interesting multifunctional carriers, capable of augmenting the cell internalization and delivering their cargo at the site of action. Doxorubicin-loaded glutathione redox-sensitive dual bio-responsive mesoporous silica nanoparticles (Dox-loaded MSN-SS-HA) surface-decorated with HA through disulfide bonds were developed by Zhao and co-workers in 2015. The drug release from these NPs was improved in the presence of glutathione, due to the cleavage of the disulfide bonds (significantly larger quantities of glutathione are present intracellularly, compared to extracellularly, especially in cancer cells), and hyaluronidases, which degrade HA into smaller fragments. The targeting functionality of HA is not affected because the degradation occurs only intracellularly. The internalization mechanism was CD44 receptor-mediated endocytosis and the NPs were more cytotoxic towards HCT-116 cells (CD44-overexpressing cancer cells) than towards NIH-3T3 cells (CD44-negative cancer cells) due to increased cellular uptake [169]. Recently, the molecular weight of HA, its influence upon the biological activity and physicochemical properties of HA conjugates and HA-decorated NPs, as well as upon the biodistribution of the carrier in vivo has come into the focus of research [170–171]. Some of the limitations can be successfully overcome by the design of multifunctional nanoscale carriers.

There is no doubt that HA improves internalization through interactions between CD44-receptor and ligands. However, the expression of HA recycling receptors (HARE and LYVE-1) in the mammalian liver [172] may negatively influence the circulation time, reducing the healing potential of targeted NDDSs. In contrast, PEGylation of HA-decorated NPs will influence the internalization potential of the nanoscale systems. Zhong et al. developed an interesting multifunctional system capable of providing the EPR effect and improved receptor–ligand interaction. They synthesized a hydroxypropyl-β-cyclodextrin (HPCD)-grafted HA polymer (HA-CD) and a pH-responsive adamantane–PEG conjugate (AD-B-PEG) with a benzoic imine linkage, and prepared Dox-loaded PEG-modified HA-CD nanoparticles through emulsion solvent evaporation. The Dox-loaded HA-based transformable supramolecular nanoplatform contains an acidity-sensitive PEG shell that can be detached in the extracellular environment of tumors, which is achieved by a benzoic imine linkage. After the intratumor localization, the NPs will transform into a “recognition” state in the acidic tumor microenvironment, exposing the HA chains at the surface and contributing to improved internalization through CD44 receptor–ligand interaction [170].

Shen et al. designed erlotinib-loaded human serum albumin–hyaluronan (ERT-HSA-HA) NPs. The NPs showed a highly efficient uptake in A549 cells and a superior antiproliferative effect. The pharmacokinetic parameters (blood residence time) in vivo of HA-decorated NPs were similar to that of ERT-HSA NPs. However, in terms of in vivo antitumor activity, mice treated with ERT-HSA-HA NPs showed a significantly suppressed tumor growth and no relapse after 30 days of treatment [173].

**Figure 6:**
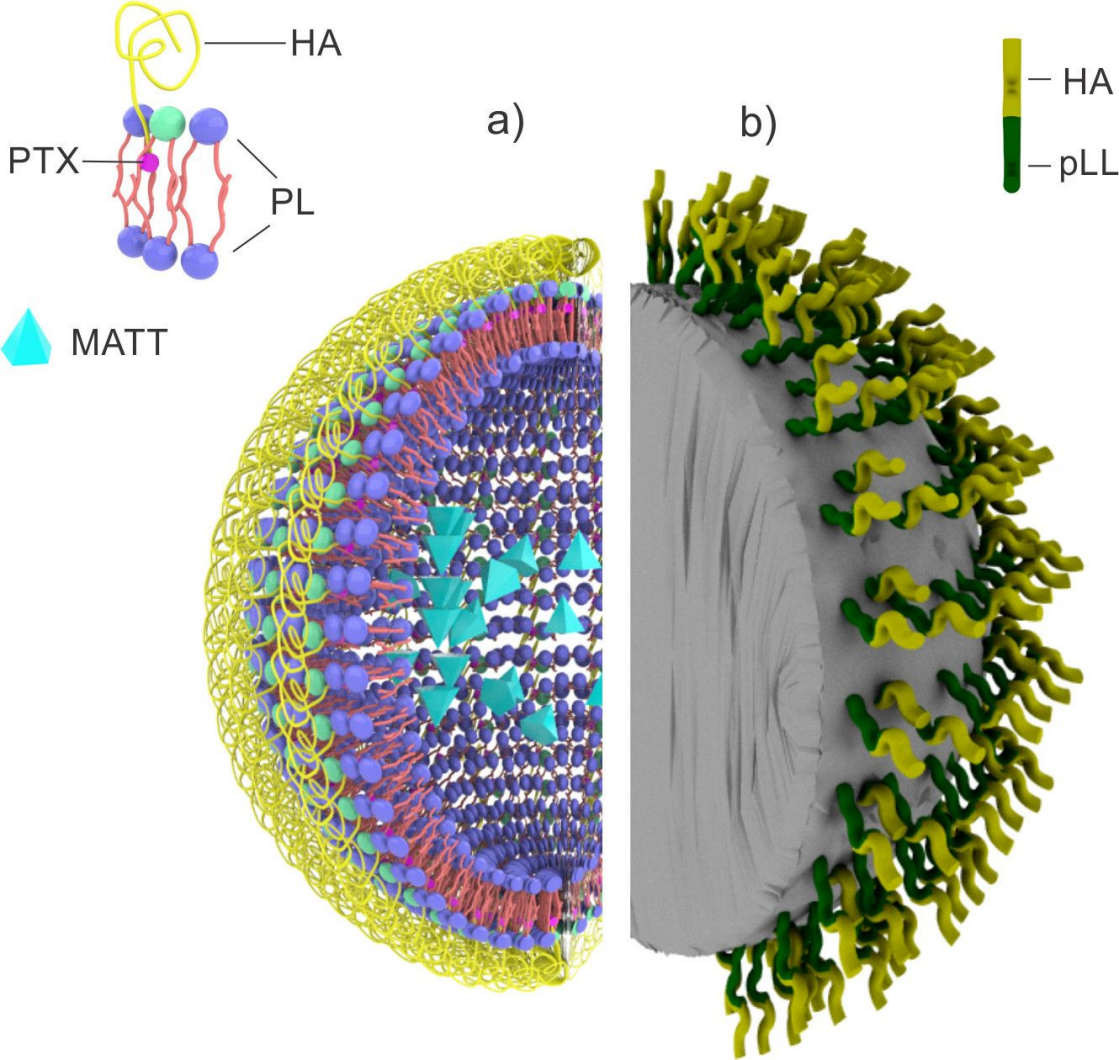
HA-functionalized NDDS with multimodal targeting capabilities. (a) HA–paclitaxel (PTX) complex and marimastat (MATT)-loaded liposomes providing dual action after thermally induced disassembly of the formulation. MATT stabilizes the tissue microenvironment by inhibiting MMPs, while HA promotes CD44-mediated uptake of PTX (PL: phospholipids); adapted from [174]. (b) HA–poly(ʟ-lysine) (pLL) layer-by-layer polystyrene nanoparticles with dual targeting capability based on hypoxia-induced surface polarity shift and HA–CD44 specific affinity; adapted from [185].

Recently, Lv et al. designed a system for breast cancer treatment equipped to target the tumor microenvironment and the cancer cells (Figure 6a). The authors recommended the administration of a prodrug, that is, a HA–PTX complex, and marimastat (MATT)-loaded thermosensitive liposomes (LTSLs) (MATT-LTSLs) for the targeting of tumor microenvironment and cancer cells. The HA–PTX complex self-assembles at the surface of the liposomes, forming hybrid nanoscale systems. Once hyperthermia is applied on the thermosensitive liposomes, the tumor environment modulator MATT, a broad-spectrum synthetic enzyme inhibitor, and the prodrug will be rapidly released and HA–PTX will be quickly internalized in the cancer cells through CD44–HA affinity. Paclitaxel will exert its activity in cancer cells and MATT will maintain the extracellular matrix integrity by inhibiting expression and activity of matrix metalloproteinases (MMPs), blocking fibroblast activation, and suppressing the degradation of the extracellular matrix. This hybrid nanosystem should be able to provide dual action and induce significant cytotoxicity and metastasis inhibition. The authors described an increased biodistribution of the NPs in the solid tumor as well as improved antitumor efficacy of the hybrid NPs in vivo [174].

Park et al. decorated cationic polyethylenimine (PEI) with anionic HA to deliver genes more efficiently into human mesenchymal stem cells (hMSCs). They showed that, through HA–CD44 interaction, PEI/pDNA complexes with HA ligands were readily internalized by HeLa cells and hMSCs. The internalization was reduced by a pretreatment with a specific monoclonal antibody that blocked CD44. Following internalization of the SOX9 gene, chondrogenesis of hMSCs was increased [175].

Recently, several studies on the pharmacological activity and the anticancer effect of triptolide (TP) revealed its molecular targets and recognized the potential to block the HA–CD44/RHAMM signaling axis. Triptolide, extracted from *Tripterygium wilfordii* celastraceae plants, is a highly effective natural anticancer drug with novel anticancer effects evaluated in many different preclinical studies [176–179]. It is also tested in phase-I clinical trials in patients with advanced gastrointestinal tumors [180]. Although it has been proven that TP inhibits the growth of multiple cancer cells, including both hematologic malignancies and solid tumor cells at low concentrations, several challenges hamper its clinical application. Among them are its high toxicity, low water solubility, and the fact that its therapeutic targets and molecular mechanisms of action are largely still under investigation [181–182]. Nanotechnology and tumor targeting may offer solutions for high toxicity and low solubility. However, for the design of a highly efficient system a detailed understanding of the therapeutic targets and signaling mechanisms affected by the drug is required. Recently, the potential of TP to block HA–CD44/RHAMM signaling and suppress the development of lung cancer was evaluated [183]. It has been concluded that there is a strong evidence of TP involvement in the alteration of the HA–CD44/RHAMM signaling axis. Several NSCLC cell lines, that is, A549, H1299, H520, and H1975, with high secretion of HA, CD44, and RHAMM were used in the study (expressed at gene and protein levels with the exception of H1975 cells, which do not express CD44). BEAS-2B bronchial cells were also used, characterized by low secretion of HA and high expression of HA synthase 1 (HAS1), compared to NSCLC. All cell lines showed high expression of HAS2 and HAS3 isoforms. Suppression of HA synthesis was evaluated through HAS2 and HAS3 expression levels and HA secretion. The effects on the CD44 and RHAMM signaling axes were measured by the expression of CD44 and RHAMM at both gene and protein levels. Further, the authors also aimed to evaluate whether the pro-apoptotic and anti-proliferative effect of TP is mediated by inhibition of HA synthesis. Therefore, they treated the cells with TP and TP with HA. TP suppressed the mRNA levels of HAS2 and HAS3 after 6 and 12 h, respectively, reduced the expression of CD44, RHAMM, EGFR, ERK, and Akt, and activated caspase 3 and PARP cleavage. Additionally, concomitant treatment with TP and HA markedly attenuated the effect of TP on these proteins. Authors also performed a proliferation and self-renewal test with TP and CD44 siRNA on putative lung cancer stem cells and confirmed their results with NSCLC cells comparing the effect of TP to the effects of transfection of NSCLC cells with HAS2, CD44, or RHAMM siRNA. Further, they incorporated the drug in liposomes and evaluated the efficacy in vitro and in vivo using an orthotopic lung tumor in a nude rat model. The combination therapy of TP in conjunction with EGFR TKIs as a long-term anticancer therapy was also suggested. The authors used a liposomal dispersion in order to point out the importance of advanced drug delivery systems for improving the efficacy and safety of TP. However, more sophisticated multifunctional TP nanoscale carriers need to be designed. In conclusion, these findings comply with the results in the literature for (i) the importance of HA–CD44/RHAMM axis targeting in lung cancer treatment, (ii) the efficacy of TP in these attempts, and (iii) the need for the design of more sophisticated multifunctional targeted drug delivery systems with combined ECM and cancer cell targeting. Multifunctional nanoscale carrier platforms based on the current knowledge about the complexity of the disease at the morphological and molecular levels, about tumor mutational signatures, about drug–disease interactions, and about cellular responses to therapy will have a huge impact and will add value to standard approaches for the design of targeted drug delivery systems.

Approaches based on network pharmacology, which integrate drug target prediction, drug–disease network analysis, and key network target screening, have been used for the prediction of the multi-targeting capabilities of compounds such as TP. Zhang et al. used a network pharmacology-based approach to predict TP targets and the mechanism of action in hepatocellular carcinoma [181]. They identified that putative TP targets were mostly associated with apoptosis signaling pathways, including crucial components in the apoptotic signaling pathway such as TNF, NFKB1, NFKBIA, RELA, BCL2, and XIAP. Further, the authors designed galactosylated chitosan (GC)-TP-NPs for hepatoma targeting, using galactose groups as specific adhesive ligands to the asialoglycoprotein receptor at the surface of hepatocellular carcinoma cells. They compared the effect on the efficacy of TP in vitro, using the HCC human cell line, and in vivo, using an SMMC-7721tumor-bearing nude mouse model. GC-TP-NPs exerted the same pro-apoptotic and anti-proliferative effects on HCC cells in vitro by inhibition of apoptosis via blocking TNF/NF-κB/BCL2 signaling, and displayed a higher efficacy in reducing tumor sizes in vivo compared to TP.

Song et al. designed NPs for dual-receptor targeted cancer therapy. Instead of using two separate localization units, the authors aimed to design an amphiphilic conjugate of methotrexate–hyaluronan–octadecylamine (MTX-HA-OCA) for curcumin (CUR) encapsulation within the hydrophobic core during self-assembly (MTX-HA-OCA/CUR NPs). Due to the structural similarity with folic acid, MTX in this system can be successfully utilized for targeting the folate receptor. MTX-HA-OCA/CUR NPs exhibited a significantly higher cell killing ability against HeLa cells than free CUR, free MTX, a CUR/MTX mixture, and HA-OCA/CUR NPs at the same concentration of CUR or MTX. They also improved tumor localization during in vivo murine tumor model studies [184].

Layer by layer (LbL) nanoparticles have attracted attention because of the ability to accommodate several targeting modalities and operate through different targeting mechanisms at the level of systemic circulation, tumor environment, and tumor cells, or to even deliver multiple agents at different targeting sites. LbL nanoparticles that show improved cell internalization due to selective interaction with CD44 cell surface receptors as well as tumor-responsive pH-induced cell drug delivery were developed by Dreaden and co-workers. The nanoscale systems were prepared by deposition of a weak polyamine, (i.e., poly(ʟ-lysine)), and a weak polyacid (i.e., hyaluronan) to create a polyelectrolyte complex bilayer, which serves as functional component of this dual-targeting LbL drug carrier (Figure 6b). The layers were assembled on fluorescent polystyrene nanoparticles via sequential adsorption and centrifugation from solutions of aqueous hyaluronan and poly(ʟ-lysine). After EPR effect-induced targeting, the hypoxic pH value of the tumor environment will induce changes at the nanoparticle surface, such as swelling and loss of anionic charge of the HA. The authors indicated that these changes improve targeting of hypoxia regions as well as cell internalization. Increased hypoxia targeting was noticed in vitro and in vivo. Apart from the significant contribution of the CD44–HA receptor–ligand interaction to internalization, the response to the hypoxic pH value and a slight increase in hydrophobicity of these nanoparticles induced the nonspecific uptake as additional internalization mechanism within the local tumor microenvironment [185].

Morton et al. applied different layer-by-layer architectures to native PLGA NPs in order to improve the pharmacokinetic properties of the nanomedicines, reduce the bolus release of the drug from the nanoparticles, and enhance the safety and circulation half-life of the drug in vivo. An improved biodistribution of drug and carrier and an increased control of the drug release rate were achieved using biomimetic alternatives to poly(ethylene glycol), specifically alginate and HA, as terminal layers for the NP delivery vehicle [186].

Novel mitochondrial and CD44 receptor dual-targeting redox-sensitive multifunctional micelles based on oligomeric hyaluronan (oHA) were proposed by Wang and co-workers. The nanoscale carrier was prepared using (5-carboxypentyl)triphenylphosphonium bromide (TPP, mitochondria-targeting moiety), oligomeric hyaluronan (oHA, hydrophilic corona and CD44-targeting moiety), disulfide bonds (S-S, linker), and curcumin (CUR, active substance and hydrophobic core), in the form of TPP-oHA-S-S-CUR micelles. First, the polymer–drug conjugate was synthesized. Then, additional curcumin was loaded into the TPP-oHA-S-S-CUR micelles via self-assembly in water. Disulfide bonds are redox-sensitive and can be broken in the presence of glutathione. This multifunctional nanoscale carrier exhibited improved stability, increased blood circulation time, and it could achieve better tumor targeting due to HA and TPP, as well as disulfide bond degradation and release of active substances in the tumor cell [187].

#### Future perspectives in the design of nanomedicines for molecular targeting of solid tumors

Molecular profiling of solid tumors and screening for cancer-specific signatures provide an opportunity to develop targeting agents for early detection and diagnosis, and to select the most effective treatment options. Chimeric antibodies, recombinant proteins, and synthetic polypeptides have emerged as excellent candidates for specific cancer targeting strategies. Coupled to nanoscale carriers, they will facilitate the delivery of their cargo to the site of action, thus allowing for better tumor imaging and increased efficacy of chemotherapy with reduced adverse effects. In the near future, predictive models and libraries will be further developed to facilitate the selection of components and predict the interaction of the designed targeted drug delivery systems with the biological and tumor environments. Also, the design of the nanoscale carriers needs to follow novel trends of molecular screening and enable the introduction of multiple targeting ligands needed for robust and specific interactions with the targeted cell populations.

Multifunctionality and/or multimodality of the nanoscale systems is a key requirement for the successful combination of several targeting components in one nanomedicine with improved targeting potential, efficacy, and safety. Arranging different modalities with environmentally triggered disassembly in vivo will enhance the ability to overcome different barriers. Equipped with several environmentally responsive tools, these nanoscale systems can achieve longer systemic circulation times, reduced non-specific uptake by the liver and spleen, low off-target delivery, fast receptor-mediated targeting, and enhanced internalization in the tumor cells. Moreover, the discoveries in the field of molecular biology of cancers and the synthesis of ligands with high avidity for the overexpressed cancer cell receptors provide promising tools for the development of highly efficient personalized medicines, designed according to the molecular cancer signature of the individual patient.

## Conclusion

Nanomedicine as a precision chemotherapy tool has been continuously evolving in the last three decades. The obvious drawbacks of the first generation of NDDSs point out the need of radical improvements in the design and performance of the carriers. As the understanding of molecular characterization of cancer progressed, the design of NDDSs evolved beyond the passive targeting based on morphological characteristics of the tumor tissue and its vasculature to a design approach based on the unique molecular signatures of the tumor niche, the cell surface, and intracellular pathways. We have presented a literature overview of NDDS design strategies for targeting myeloid leukemia and EGFR/CD44-positive solid tumors. In both cases, the design of multifunctional nanoscale carriers coupled with advances in molecular targets for cancer therapy, and the advances in predictive models and libraries for the selection of ligands with high affinity towards molecules from the tumor microenvironment are contributing to the future development of targeting NDDSs. Alongside with the system multifunctionality, the modeling of the behavior of bioresponsive targeting modalities arranged in one nanosystem in different biological environments, as well as predictive models for nanoscale biointeraction will put on the horizon the next generation of nanomedicines.
